# Bacterial polysaccharide lyase family 33: Specificity from an evolutionarily conserved binding tunnel

**DOI:** 10.1073/pnas.2421623122

**Published:** 2025-02-11

**Authors:** Mélanie Loiodice, Elodie Drula, Zak McIver, Svetlana Antonyuk, Arnaud Baslé, Marcelo Lima, Edwin A. Yates, Dominic P. Byrne, Jamie Coughlan, Andrew Leech, Shahram Mesdaghi, Daniel J. Rigden, Sophie Drouillard, William Helbert, Bernard Henrissat, Nicolas Terrapon, Gareth S. A. Wright, Marie Couturier, Alan Cartmell

**Affiliations:** ^a^Université Grenoble Alpes, CNRS, Centre de Recherche sur les Macromolécules Végétales, Grenoble 38000, France; ^b^Architecture et Fonction des Macromolécules Biologiques, Aix-Marseille Univ, CNRS, Institut National de Recherche pour l'Agriculture, l'alimentation et l'Environnement, Marseille 13288, France; ^c^Biotechnologie et Biodiversité Fongiques, Institut National de Recherche pour l'Agriculture, l'Alimentation et l'Environnement, Marseille 13288, France; ^d^Department of Biology, University of York, Heslington, York YO10 5DD, United Kingdom; ^e^Department of Biochemistry, Cell and Systems Biology, Institute of Systems, Molecular and Integrative Biology, University of Liverpool, Liverpool L69 7ZB, United Kingdom; ^f^Newcastle University Biosciences Institute, Medical School, Newcastle University, Newcastle upon Tyne NE2 4HH, United Kingdom; ^g^School of Life Sciences, Keele University, Keele, Staffordshire ST5 5BG, United Kingdom; ^h^Department of Biology, Technology Facility, University of York, Heslington, York YO10 5DD, United Kingdom; ^i^Computational Biology Facility, MerseyBio, University of Liverpool, Liverpool L69 7ZB, United Kingdom; ^j^Department of Biological Sciences, King Abdulaziz University, Jeddah 23218, Saudi Arabia; ^k^Department of Biotechnology and Biomedicine (DTU Bioengineering), Technical University of Denmark, Kgs. Lyngby DK-2800, Denmark; ^l^School of Life Sciences, University of Essex, Colchester CO4 3SQ, United Kingdom; ^m^York Structural Biology Laboratory, University of York, York YO10 5DD, United Kingdom; ^n^York Biomedical Research Institute, University of York, York YO10 5DD, United Kingdom

**Keywords:** structural biology, tunnel topography, glycosaminoglycans, conformational change, modeling

## Abstract

Acidic glycans are essential for the biology of multicellular eukaryotes. To utilize them, microbial life including symbionts and pathogens has evolved polysaccharide lyases (PL) that cleave their 1,4 glycosidic linkages via a β-elimination mechanism. PL family 33 (PL33) enzymes have the unusual ability to target a diverse range of glycosaminoglycans (GAGs), as well as the bacterial polymer, gellan gum. In order to gain more detailed insight into PL33 activities we recombinantly expressed 10 PL33 members derived from all major environments and further elucidated the detailed biochemical and biophysical properties of five, showing that their substrate specificity is conferred by variations in tunnel length and topography. The key amino acids involved in catalysis and substrate interactions were identified, and employing a combination of complementary biochemical, structural, and modeling approaches, we show that the tunnel topography is induced by substrate binding to the glycan. Structural and bioinformatic analyses revealed that these features are conserved across several lyase families as well as in mammalian GAG epimerases.

## Significance

Acidic glycans, containing uronic acid sugars and sulfates, are essential for multicellular eukaryotic organisms. Despite eukaryotes being the main producers, microbes are their principal degraders, deploying specific polysaccharide lyase (PL) enzymes to enable bacteria, as well as viruses and fungi, to utilize them. Here, we analyze the structural and mechanistic features of PL family 33 revealing its close relationship to several other PL families, but also to mammalian glycosaminoglycan epimerases that modify the substrates they degrade.

Complex glycans containing uronic acids are ubiquitous in multicellular eukaryotes. In plants, pectins, which are complex glycans containing D-galacturonic acids (1, 2), are major components of the primary cell wall of dicotyledonous plants (3) and are implicated in flowering (4). Alginate is a mixed linkage polymer of D-mannuronic acid and its C-5 epimer L-guluronic acid found in the cell walls of brown seaweed and has numerous applications in the food (5), pharmaceutical (6), and biofuel industries (7). The extracellular matrix of mammals contains several glycosaminoglycans (GAGs): heparan sulfate (HS), chondroitin sulfate (CS), dermatan sulfate (DS), and hyaluronic acid (HA). These contain either D-glucuronic acid and/or its C-5 epimer L-iduronic and are essential for cell signaling, growth, and development (8–10). GAGs are also high-priority nutrient sources for symbiotic *Bacteroides spp.* of the mammalian distal colon (11, 12). Although less common, uronic acid–containing complex glycans are also components of bacterial capsular structures and exopolysaccharides, including K5 from *Escherichia coli* K5 [which shares the same sequence as heparosan (13)] and gellan gum which is produced by *Sphingomonas elodea* (14).

**Fig. 1.**
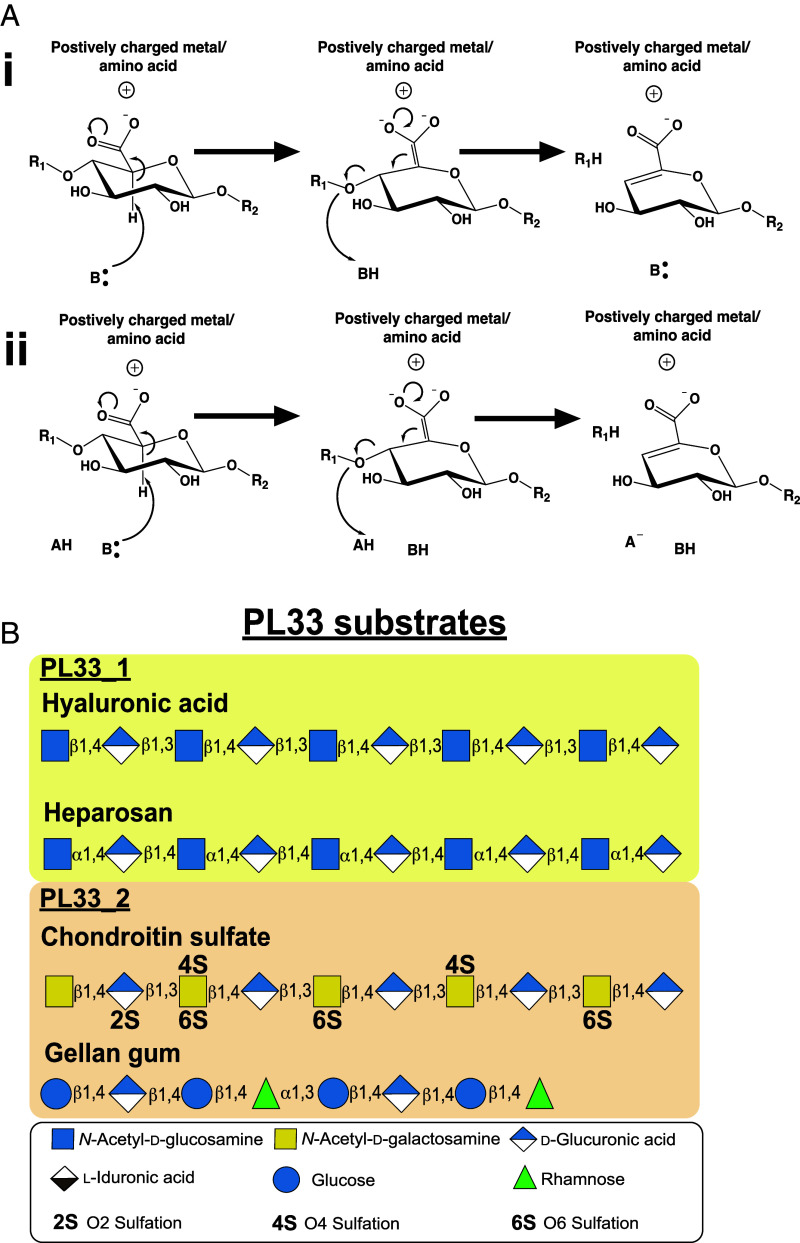
General lyase mechanism and PL 33 substrates. (*A*) General mechanisms for β-elimination by polysaccharide lysase enzymes. R_1_ and R_2_ denote hexosamine sugar residues; (i) demonstrates a single acid/base catalytic residue, which both abstract a C5 proton and donate a proton to the new reducing end sugar, R_1_ in an asynchronous manner; E1cb elimination (ii) demonstrates a mechanism that utilizes a general base, to abstract a C5 proton and a general acid, to protonate the new reducing end sugar, R_1_ in a synchronous manner; E2 elimination. The stabilizer can be a metal or an amino acid depending on the lyase family; (*B*) The structures of the PL33 substrates and which subfamily they are targeted by.

In multicellular eukaryotes, glycoside hydrolases catalyze the hydrolysis of uronic acid–containing complex glycans (15). In bacteria, however, a different strategy employing a class of enzyme known as polysaccharide lyases (PL) is deployed (16, 17). PLs selectively target glycans composed of uronic acids with a C4 hydroxyl group linked to the preceding sugar in the glycan chain. This enables PLs to operate through a β-elimination mechanism that relies on proton abstraction from the C5 carbon of the uronic acid sugar ring, resulting in double bond formation between C4 and C5 and elimination of the preceding sugar (18) (Fig. 1*A*).

PL are currently cataloged in the carbohydrate-active enzyme database (CAZy) into 44 families based on their sequence homology; some of these families are further divided into subfamilies (16, 17). At present, fewer than half of PL families have had their functional, structural, and mechanistic aspects interrogated, leading to a significant gap in our understanding of PL evolution and function (16, 17). Initially, a single member of PL family 33 (PL33) was identified as a HA/CS targeting enzyme (19, 20). Other members have since been shown to be specific for CS and gellan gum (21), while in this work, we identify members targeting heparosan. HA and CS (Fig. 1*B*) are both composed of a repeating disaccharide unit of D-glucuronic (GlcA) acid β1,3 linked to an amino sugar (*N*-acetyl D-glucosamine [GlcNAc] in HA and *N*-acetyl D-galactosamine [GalNAc] in CS) (20). These disaccharides are then linked together via β1,4 glycosidic bonds. The complexity of the CS glycan chain is increased further through selective sulfation of *O*4 and *O*6 of GalNAc and *O*2 of GlcA (22). HA has no further glycan complexity beyond its disaccharide repeat but can reach sizes in the MDa range (23). Heparosan, also undecorated, is a polymer comprising [-4-*N*-acetyl D-glucosamine-α1,4-D-glucuronic acid-β1-] repeats (Fig. 1*B*). By contrast to the disaccharide repeat of GAGs, gellan gum (Fig. 1*B*) is composed of a repeating tetrasaccharide of D-glucose (β1,4) D-glucuronic (β1,4) D-glucose (β1,4) L-rhamnose (α1,3) (24).

PL33 is divided into two subfamilies: Members of PL33_1 have been shown to preferentially degrade HA and heparosan (this work), while those from PL3_2 target CS and gellan gum. Detailed mechanistic and structural data are lacking for PL33, and its catalytic apparatus are unknown. To address this dearth of information, we report detailed biochemical analyses of five PL33 enzymes from diverse sources. Three belong to PL33_1 and two belong to PL33_2. The three characterized PL33_1 enzymes were all from *Bacteroides* species inhabiting the human colon: BT4410 (*Bt*PL33^HA^) and BcellWH2_04512 (*Bc*PL33^HA^) are hyaluronidases from *Bacteroides thetaiotaomicron VPI-5482* (*B. theta*) and *Bacteroides cellulosilyticus WH2* (*B. cell*), respectively, while HMPREF1181_01744 (*Bs*PL33^Heparosan^) is a heparosanase from *Bacteroides stercoris CC31F* (*B. stercoris*). Both PL33_2 enzymes, OPIT5_09595 (*Ob*PL33^gellan^) and OPIT5_11155 (*Ob*PL33^CS^), are from *Opitutaceae bacterium TAV5* (*O. bacterium*), isolated from the hindgut of termites, and are active against gellan gum and CS, respectively.

The apo and substrate-bound crystal structures of two PL33_1 members, *Bt*PL33^HA^ and *Bc*PL33^HA^, combined with mutagenic, kinetic, and biophysical analyses, reveal the critical residues for catalysis and substrate recognition in this family. Furthermore, we employ a combination of size exclusion chromatography (SEC)-coupled light-scattering, small-angle X-ray scattering (SAXS), AlphaFold2 (AF2) modeling, and molecular dynamic simulations to define the oligomeric state and structural changes of these enzymes in the absence and presence of substrate. The findings suggest that conformational changes are driven by catalysis and that key features observed among PL33 members are conserved across a broader evolutionary landscape that extends not only to other PL but also to mammalian epimerases. Our data demonstrate the power of a combined structural biology (X-ray crystallography, SAXS) and AI approach (AF2) to delineate protein dynamics. Neither technique alone was able to capture the biological reality of the PL33 catalytic process.

## Results

### Phylogenetic and Biochemical Analysis of PL33 Enzymes.

**Fig. 2.**
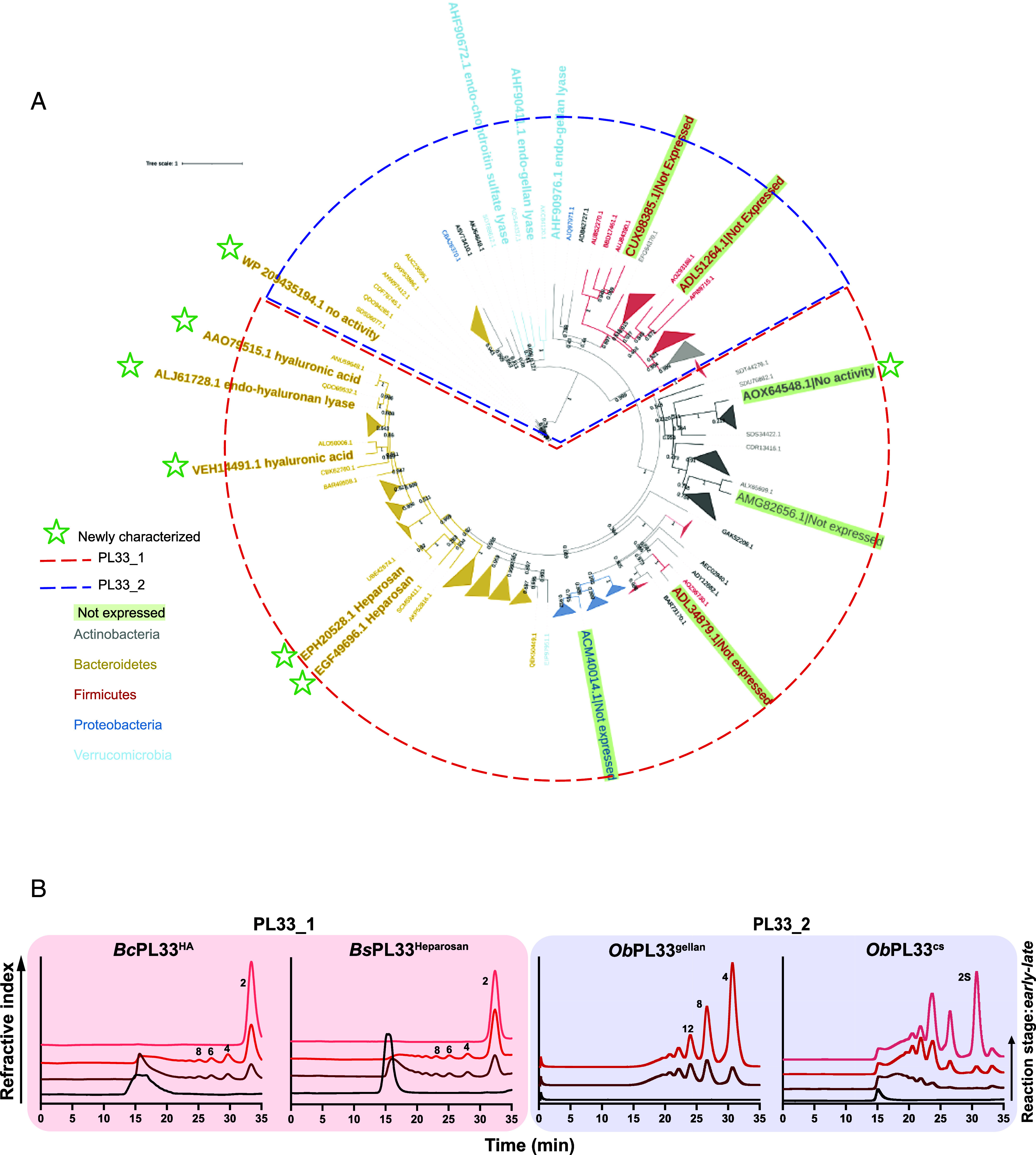
Biochemical activities of PL33 across its two subfamilies. (*A*) Phylogenetic tree of PL33 family showing subdivision into subfamilies 1 and 2 highlighted by the red and blue dashed line, respectively. Enzymes characterized in this work are labeled with green stars. Enzymes for which no soluble protein could be obtained are indicated by a black dot. Major taxonomic groups were color-coded (tree branches and leaf labels): red for Firmicutes, bright blue for Proteobacteria, light blue for Verrumicrobia, gray for Actinobacteria, and brown for Bacteroidetes. (*B*) Analysis of the mode of action of PL33 representatives. For each of the four substrates targeted by PL33 enzymes, degradation products were monitored using size-exclusion chromatography. DP: degree of polymerization is indicated by a number with 2 being a disaccharide, 4 a tetrasaccharide, etc.

PL33 divides into two distinct subfamilies. A distance tree was built from 462 catalytic domain sequences and confirmed a clear subdivision into PL33_1 and PL33_2 subfamilies (Fig. 2*A*). PL33_1 comprises sequences mainly from the Bacteroidota, Proteobacteria, and Actinobacteria phyla, whereas subfamily PL33_2 is composed mainly of sequences from the Bacillota phylum. Of the PL33 sequences currently cataloged in the CAZy database, ~85% were isolated from the terrestrial biosphere (49% land/soil, 28% human body, and 8% land animals), and 12% isolated from a marine environment. Comparing PL33_1 to PL33_2, there is a marked increase in PL33_2 sequences from marine (9 to 17%) and animal (5 to 14%) environments and a decrease in those from the human body (32 to 20% (Datasets S1 and S2).

We next purified 10 PL33 members from all major environments, five from PL33_1 and five from PL33_2, while five others were not successfully expressed. Purified proteins were screened for activity against a range of uronic acid–containing polysaccharides (Fig. 2*B* and *SI Appendix*, Table S1). Of the five PL33_1 proteins, four were from *Bacteroides* species of the human digestive tract and one from the human oral cavity bacterium *Prevotella oris* (*P. oris*) (*SI Appendix*, Table S1). We could only detect activity toward heparosan from the PL33_1 enzymes from *B. stercoris* and *B. clarus,* while the *B. theta* and *B. cell* enzymes showed specificity for HA, consistent with previous reports (2, 21), but had some activity on CS. The *P. oris* enzyme displayed weak activity against HA, CS, DS, and heparosan (*SI Appendix*, Table S1). Three of the four PL33_2 proteins were from the termite hind gut bacterium *O. bacterium*. One of these enzymes cleaved gellan gum, while one had specificity for CS, but displayed some activity against DS and glucuronan. The final *O. bacterium* PL33, and PL33 from the marine bacterium *Maribacter forsetii* and from the Actinobacteria species *Curtobacterium sp.*, displayed no activity toward our panel of polysaccharides.

Five PL33 members were targeted for detailed biochemical characterization. For PL33_1, the enzymes BT4410 from *B. theta* (*Bt*PL33^HA^), BcellWH2_04512 from *B. cell* (*Bc*PL33^HA^), which have specificity for HA, and HMPREF1181_01744 from *B. stercoris* (*Bs*PL33^Hep^), which is active against heparosan, were selected. While, for PL33_2 OPIT5_09595 (*Ob*PL33^gellan^) and OPIT5_11155 (*Ob*PL33^CS^), active against gellan gum and CS respectively, were selected. A pronounced *endo*-processive mode of action was readily observable for all enzymes except *Ob*PL33^CS^ (Fig. 2*B*). *Ob*PL33^CS^ was clearly *endo*-acting but, the nature of the CS substrate prevents the clear determination of processivity. However, the ^1^H NMR spectra of reaction end products confirmed that DP2 was the final degradation product for all GAG degrading enzymes (*SI Appendix*, Fig. S1).

### Tertiary Structure of PL33 Enzymes.

**Fig. 3.**
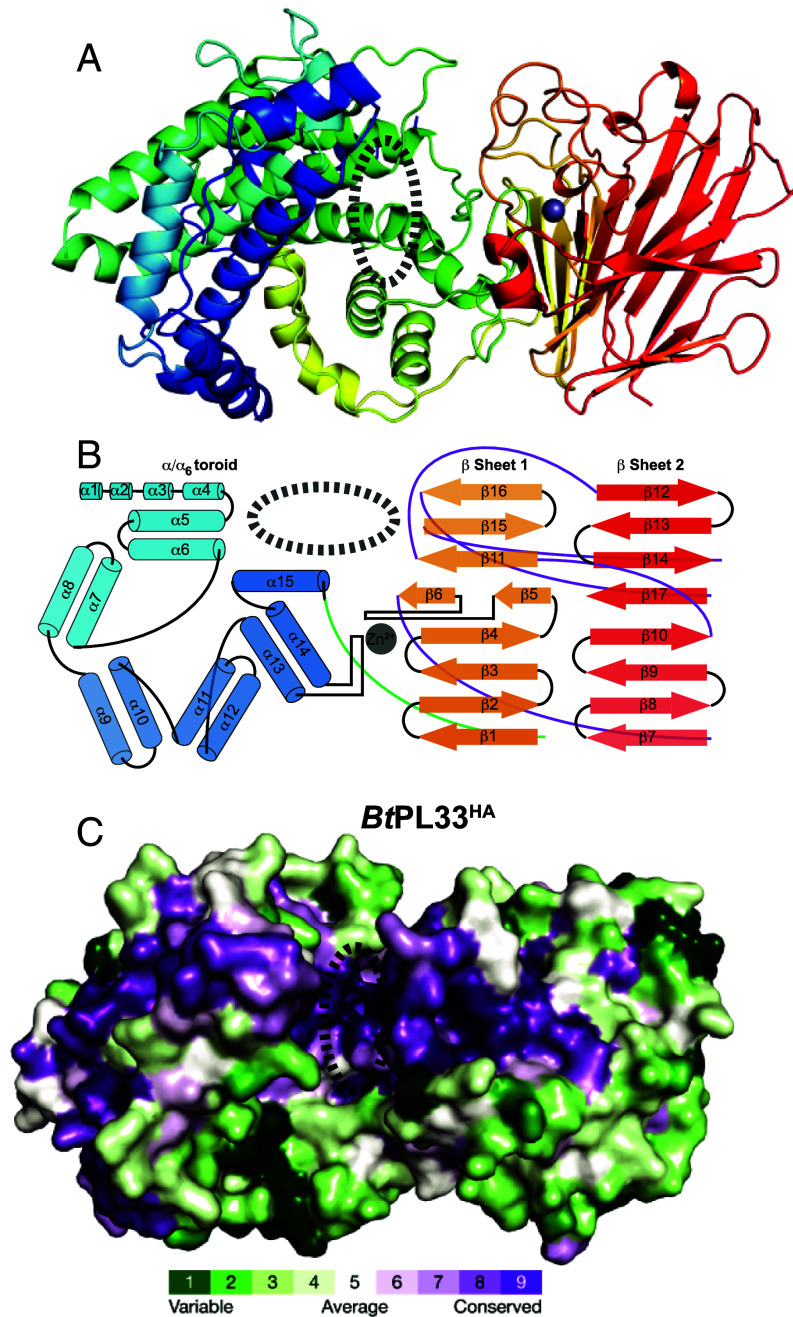
PL33 members have a two-domain structure creating a cleft. (*A*) Cartoon of *Bt*PL33^HA^ showing its domain structure graded blue to red from the N to C terminus. A dashed oval shows the location of substrate binding located primarily on the N-terminal domain; (*B*) A diagrammatic representation of the fold of *Bt*PL33^HA^. A green loop represents the connection between the N- and C-terminal domains, while a purple loop shows the interchange between β sheets 1 and 2. (*C*) A Consurf analysis of *Bt*PL33^HA^ highlighting that there is a significant accumulation of conserved residues within the cleft between the N- and C-terminal domains.

Crystal structures of *Bt*PL33^HA^ and *Bc*PL33^HA^ reveal a two-domain structure. The enzymes are 68% identical and contain a larger N-terminal α-helical (α/α)_6_ toroid domain (residues 21 to 401; *Bt*PL33^HA^ numbering used throughout this section unless specified) and a smaller β-sheet C-terminal domain (residues 402 to 635) (Fig. 3*A*).

The N-terminal domain is composed of 15 α-helices (counted as having a minimum of two turns). The first four α-helices run in series; the first two being composed of only two turns. α-helices 5 to 14 form the bulk of the (α/α)_6_ toroid with each helix running antiparallel to the next. α-helix 15, arches over 13 and 14 and leads into the C-terminal domain (Fig. 3*A*). The C-terminal domain is composed of two antiparallel β-sheets consisting of 17 β-strands that run nonsequentially. β-sheet 1 is composed of consecutive β-strands 1 to 6, together with strands 11, 15, and 16. β-sheet 2 has consecutive β-strands 7 to 10 but is then rejoined by β-strand 12 of β-sheet 1 which then runs in consecutive β-strands up to β-strand 14. β-sheet 2 is finished by β-strand 17 which threads between β-strands 10 and 14 to finalize the fold (Fig. 3 *A* and *B*).

The C-terminal domain possesses an extended loop region between β-strands 5 and 6 (Leu455-Leu484: *Bt*PL33^HA^, and Ile464-Leu493: *Bc*PL33^HA^), forming an octahedral Zn^2+^ coordination site [supported by anomalous X-ray diffraction data (*SI Appendix*, Fig. S2)], along with the loop region between α-helices 12 and 13 from the N-terminal domain. The Zn^2+^ coordination site is a conserved feature in PL33.

The two domains oppose each other in such a way that a deep, open-ended cleft is formed resembling an active site that would be compatible with an *endo*-processive mode of action of *Bt*PL33^HA^ and *Bc*PL33^HA^ (Fig. 3 *A* and *B*). Analysis using the Consurf server (25), which maps residue conservation from a sequence alignment to the structure, demonstrated a high level of amino acid conservation within the cleft, consistent with it housing the active site residues (Fig. 3*C*).

### PL33 Quaternary Structure.

Analysis of the five enzymes via SEC-coupled light scattering and SAXS indicated that the three PL33_1 enzymes *Bt*PL33^HA^, *Bc*PL33^HA^, and *Bs*PL33^Heparosan^ are dimeric, while the PL33_2 enzymes *Ob*PL33^CS^ and *Ob*PL33^Gellan^ are monomeric (*SI Appendix*, Fig. S3). The PL33_1 enzymes share a similar, but nonidentical, dimer interface (*SI Appendix*, Fig. S4 *A*–*C*). SAXS data indicate that the dimeric enzymes all bind as head-to-head homodimers, generating elongated envelopes. This is consistent with PISA (26) analysis of the crystal structures of *Bt*PL33^HA^ and *Bc*PL33^HA^ which predicted a buried surface area of ~1,388 Å^2^ and 1,006 Å^2^, and a complex significance score of 0.207 and 0.290, respectively.

### Substrate Complex Interactions and Mutagenic Analysis.

**Fig. 4.**
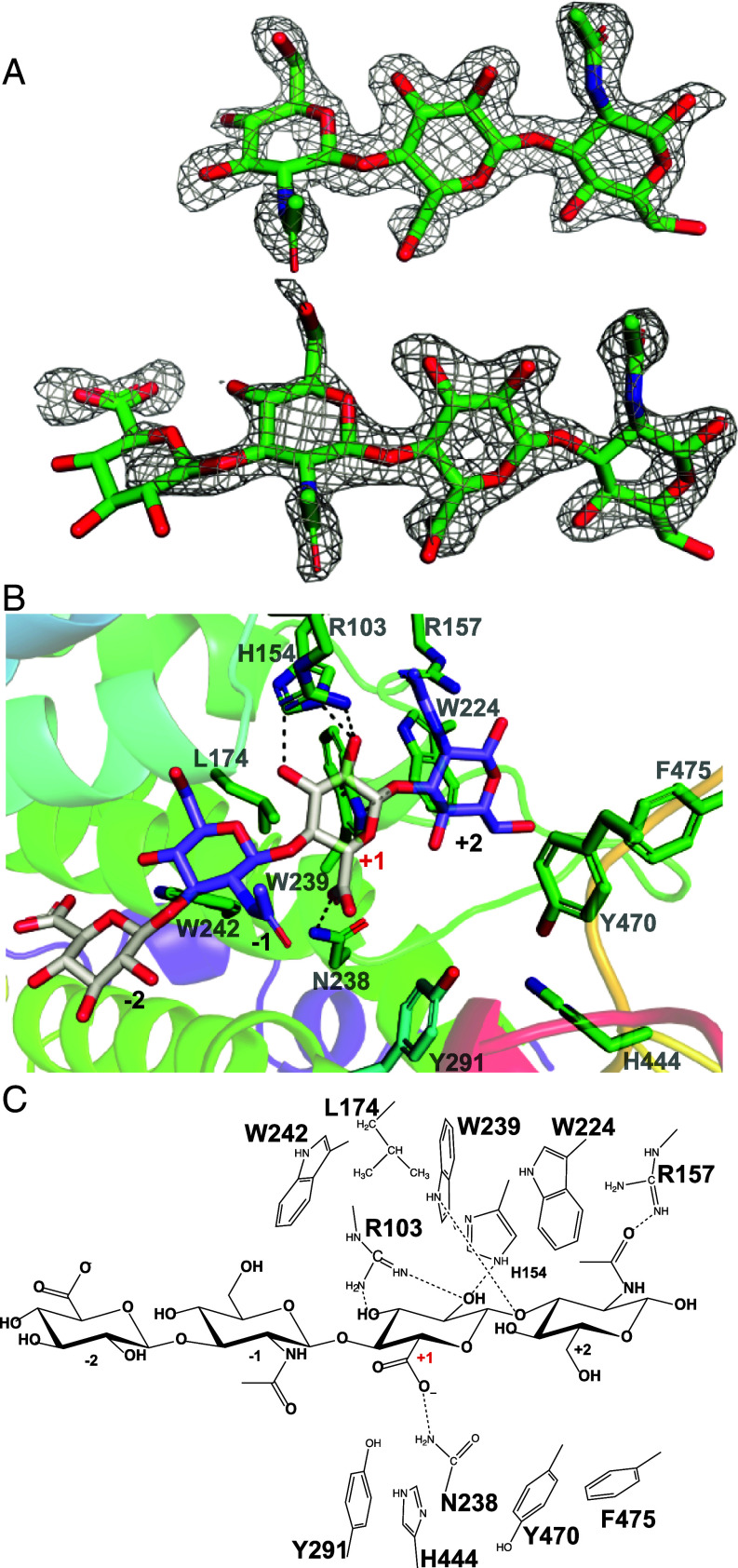
Substrate complex and active site interactions. (*A*) A weighted 1.8 Å 2m*F*_obs_-D*F*_c_ electron density map, contoured at 1σ, of the soaked hyaluronic tetrasaccharide in complex with *Bt*PL33^HA^; (*B*) Cartoon and stick representation of the active site interactions between *Bt*PL33^HA^ and a hyaluronic tetrasaccharide; (*C*) A ChemDraw representation of the active site interactions between *Bt*PL33^HA^ and a HA tetrasaccharide.

Based on the Apo structures of *Bt*PL33^HA^ and *Bc*PL33^HA^, combined with Consurf analysis (25), a total of 20 *Bt*PL33^HA^ mutants were made in order to understand the roles of individual amino acids in substrate binding and catalysis (*SI Appendix*, Table S2). This enabled the structural characterization of a catalytically compromised variant (Y291A) of *Bt*PL33^HA^ in complex with a HA tetrasaccharide. The complex contained two molecules of *Bt*PL33^HA^ in the asymmetric unit, with one molecule containing electron density for which a trisaccharide could be modeled, and the other for which a less well-ordered tetrasaccharide could be modeled (Fig. 4*A*).

A hydrophobic platform composed of W224, W239, L174, and W242 underpins binding at the +2, +1, and −1 subsites, while no obvious interactions occur with the −2 sugar (Fig. 4 *A*–*C*). Within PL33_1 W242, L174, and W239 are invariant, while at the equivalent site for W242 in PL33_2, a Val occupies this position (*SI Appendix*, Fig. S5 and Table S2). Mutation of *Bt*PL33^HA^ W242 to Ala or Val had only minor effects on catalytic efficiency (*SI Appendix*, Fig. S6*A* and Table S2). In contrast, L174A and W239A mutations resulted in a ~20 and ~100-fold drop in *k*_cat_/*K*_M_ respectively, driven by a significant increase in *K*_M_ for W239A, and a significant decrease in *k*_cat_ for L174A (*SI Appendix*, Fig. S6*A* and Table S2).

The +1 subsite, the site of catalysis, is where the majority of interactions occur.

The side chain of R103 interacts with the *O*2 and *O*3 of the +1 GlcA, while H154 only interacts with *O*2 (Fig. 4*B*). Alanine mutants of both drove significant increases in *K*_M_, resulting in 50 to 60-fold reductions in *k*_cat_/*K*_M_ (*SI Appendix*, Fig. S6*A* and Table S2). Within PL33_2, R103 and H154 are consistent in all sequences, while in PL33_1 R103 is absent in only 3 of 114 analyzed sequences (*SI Appendix*, Fig. S5). N238 interacts with the carboxylate of the +1 GlcA through its amide group, while Y291 lies ~6 Å from C5 (Fig. 4 *B* and *C*). No activity could be quantified for the mutants N238A or Y291A (*SI Appendix*, Fig. S6*A* and Table S2). Qualitative end point assays, employing both high substrate and high enzyme concentrations, demonstrated that N238A and Y291A retained residual activity, but were unable to complete the degradation of HA over a 16 h period at 37 °C (*SI Appendix*, Fig. S6 *D* and *F* and Table S2). Mutation of H444 to Ala, a residue located just over 8 Å from C5 but only ~3 Å from Y291, resulted in a ~17,000-fold reduction in specific activity (*SI Appendix*, Fig. S6*A* and Table S2) but was insufficient to completely abrogate activity (*SI Appendix*, Fig. S6*D*). However, tandem mutation of Y291A and H444A did abolish *Bt*PL33^HA^ activity (*SI Appendix*, Fig. S6*E*), with only modest changes in thermal stability (*SI Appendix*, Fig. S6 *B* and *C* and Table S3). N238, Y291, and H444 are invariant across PL33. Collectively, these data support a role for these residues as part of the catalytic apparatus. However, it should be noted that although both Y291 and H444 are positioned below the beta face of the +1 GlcA, they are too remote to directly abstract a proton from C5. Furthermore, N238 coordinates with the carboxyl group from above the alpha face, suggesting a role in charge stabilization rather than proton abstraction. This suggests significant conformational shifts are needed for Y291 and H444 to perform a direct role in catalysis.

### AF2 Modeling Reveals a Closed Conformation.

**Fig. 5.**
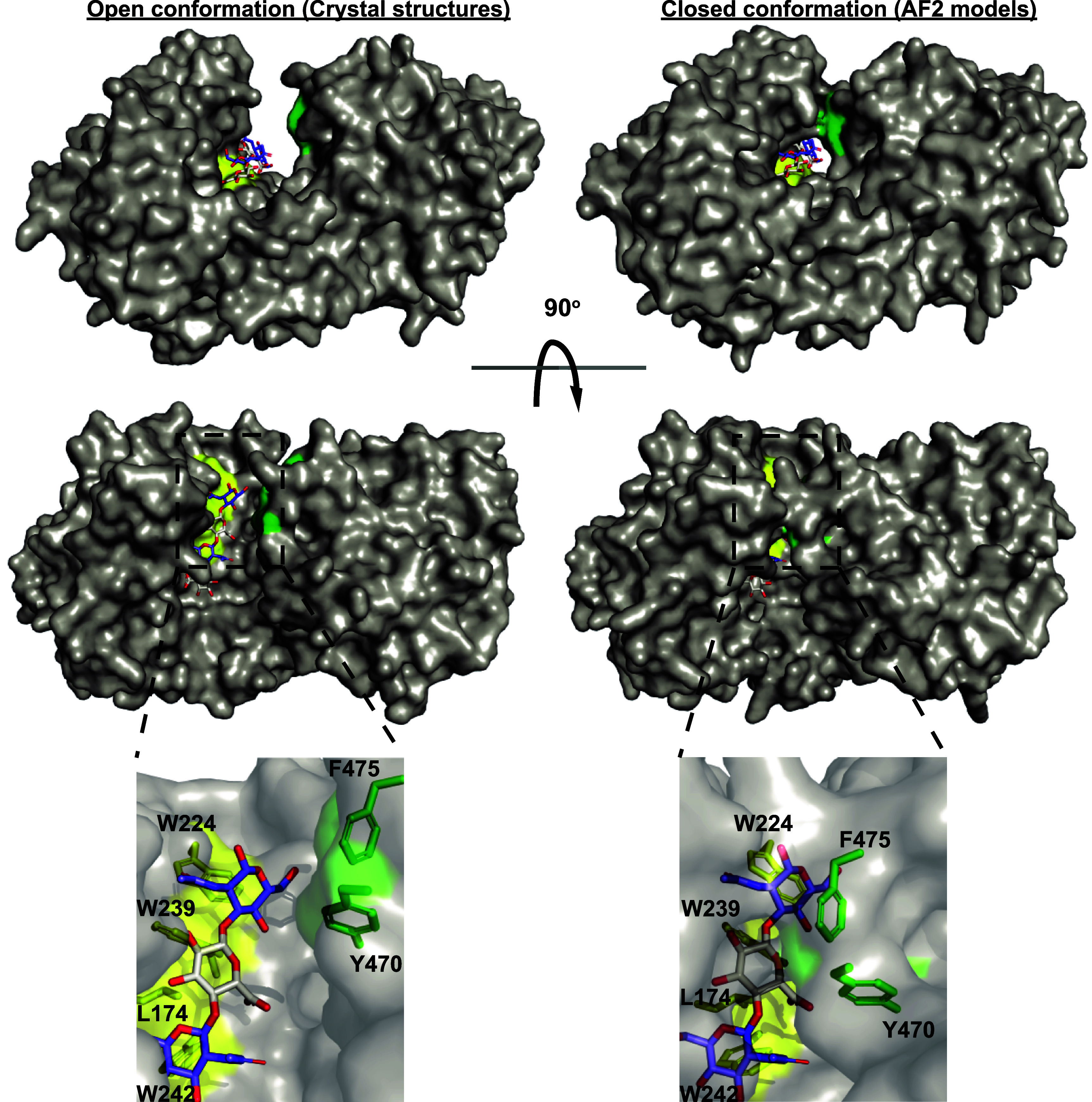
Crystallographic structures and AF2 models predict distinct conformational states *Bt*PL33^HA^. (*Left*) The open confirmation of *Bt*PL33^HA^ in complex with substrate. (*Right*) The closed confirmations generated by AF2 modeling with substrate from the *Bt*PL33^HA^ complex overlaid. Green indicates the N-terminal domains hydrophobic platform formed by W224, W239, L174, and, W242, and yellow indicates Y470 and F475 that close upon substrate binding, observed in the AF2 models.

For all five of the analyzed enzymes, AF2-generated models (27) display a closed tunnel-like topography housing the +2 and +1 subsites (Fig. 5 and *SI Appendix*, Fig. S7). The AF2 models predict that in the closed state the C-terminal loop, between β strands 5 and 6, containing the residues Y470 and F475 moves 5 to 6 Å closer to the substrate. The phenol of Y470 moves within 5 to 6 Å of the +1 GlcA and *N*-acetyl of the −1 GlcNAc, while F475 moves within 5 Å of the +2 GlcNAc, forming the tunnel topography. This differs significantly from the wide-open cleft observed in the crystal structures of both *Bt*PL33^HA^ and *Bc*PL33^HA^ (Fig. 5). Fitting of both the *Bt*PL33^HA^ and *Bc*PL33^HA^ crystal structures and their AF2 models to SAXS data indicated that the best fit was for the open conformation observed in the crystal structures (*SI Appendix*, Fig. S8 *A* and *B*). In addition, ~500,000 *Bt*PL33^HA^ dimer models, generated by molecular dynamics analysis (see below), were fitted to the experimental SAXS data. Seven models, all in the open conformation, fitted exceptionally well (chi values 1.23 to 1.24) in comparison with the *Bt*PL33^HA^ crystal structure and the best AF2 model (chi value 1.54 and 1.91) (*SI Appendix*, Fig. S8 *C*–*F*). To determine whether conformational change accompanies ligand binding, we performed a SAXS experiment with the inactive *Bt*PL33^HA^ Y291A mutant in the presence of a HA tetrasaccharide (this mutant displays residual activity on HA but cannot cleave tetrasaccharides) (Fig. 4*A* and *SI Appendix*, Fig. S6). However, neither the presence of the mutation nor the substrate induced a conformational change as indicated by unaltered scattering profiles (*SI Appendix*, Fig. S9). These data support the view that PL33 members exist in the open inactive conformation and their transition to a closed conformation is coupled to catalysis.

### Sampling of *Bt*PL33^HA^ Conformational Space Reveals Local and Global Flexibility.

**Fig. 6.**
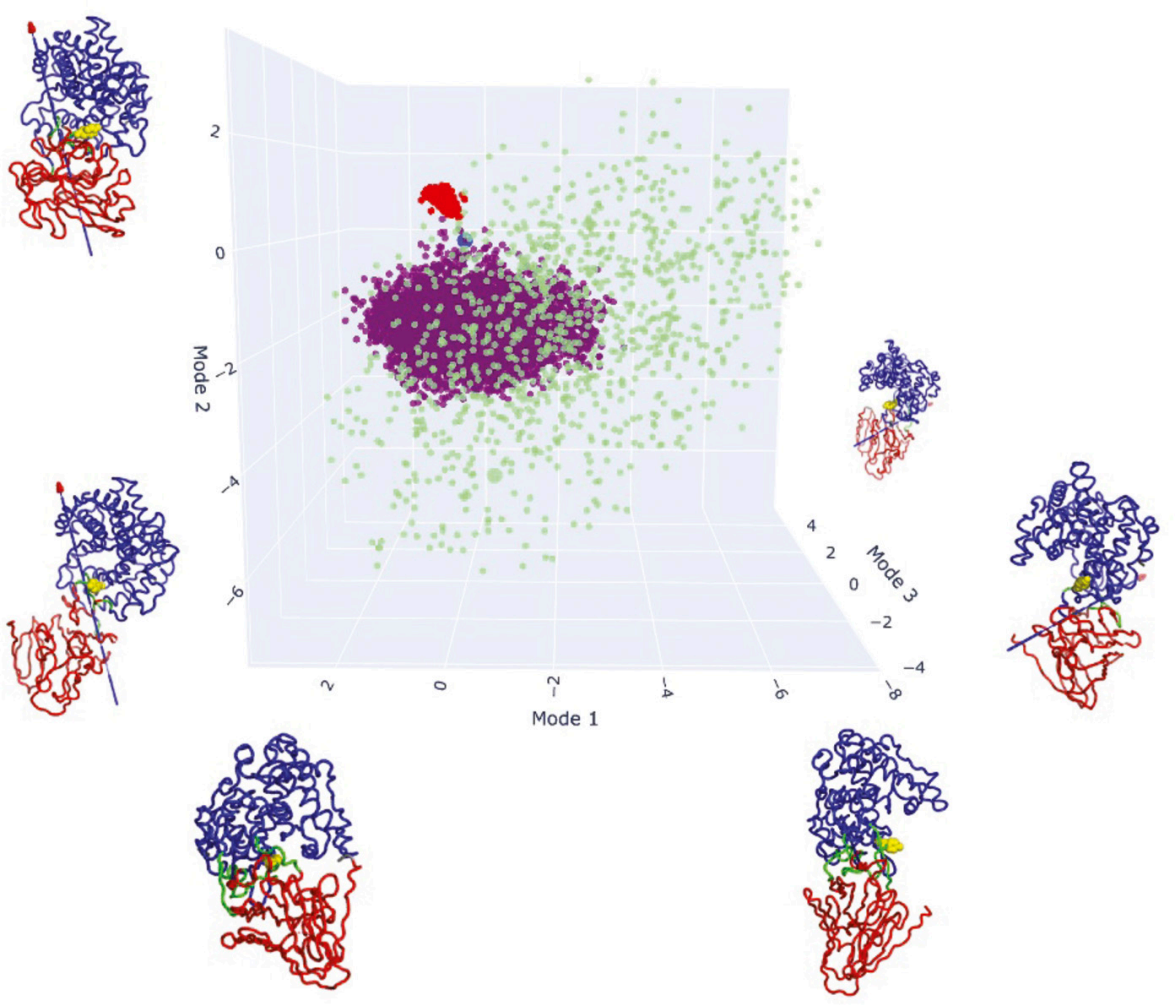
Sampling of *Bt*PL33^HA^ conformational space reveals local and global flexibility. Conformational space is represented by the top three modes identified by Eigenvector analysis and the three sets of structures are mapped: The blue point is the experimental structure, purple points are from molecular dynamics, red are AF2 models, and green are CONCOORD results. Each axis is illustrated with the maximum and minimum projections along the mode, colored according to DynDom analysis. Blue and red represent different dynamic domains, in each case corresponding largely to the (α/α)_6_ toroid and all-β domains, respectively. Hinge residues are shown in green, with the arrow representing the axis of rotation (the perspective for mode 1 is along the axis of rotation resulting in poor visibility of the arrow). Tyr291 is shown in yellow to mark the center of the catalytic site cleft.

Next, we performed molecular dynamic simulations to determine the extent of PL33 conformational plasticity, using the *Bt*PL33^HA^ crystal structure model. For this purpose, we employed a conformationally diverse AF2 approach (*Methods*) (28, 29) [8], a CONCOORD distance geometry analysis, and full-atom molecular dynamics. All three methods illustrate the flexibility of *Bt*PL33^HA^ allowing the opening and closing of the catalytic site. Eigenvector analysis was performed on the combined results with GROMACS and DynDom (30) used to characterize the maximum and minimum projected protein structures of the top three eigenvectors. For each of the top three eigenvectors the DynDom-defined dynamic domains corresponded roughly to the N-terminal (α/α)_6_ toroid and anti-parallel β sheet structural domains of *Bt*PL33^HA^ (Fig. 6). The angles of rotation determined by DynDom were substantial at 70, 73, and 70°, respectively. Notably, the top two modes each influence the shape of the cleft housing the catalytic site, the first corresponding to a general opening and closing motion, the second representing more of a transient closure of one end of the catalytic site cleft.

The CONCOORD results are the most structurally diverse, but the MD simulation samples all of the same modes of conformational variability. It should be noted, however, that the trajectory travels along the direction of opening-up of the catalytic site cleft: The simulation did not sample full reclosing which may, indeed, require substrate binding. This is illustrated by the Cβ separation between the putative catalytic Tyr291 and its potential activator His444 (*SI Appendix*, Fig. S10*A*) which is ~8.8 Å in the Apo crystal structure but which fluctuates between 12 and 24 Å during the simulation.

The conformational space explored by AF2 is much more condensed (Fig. 6). Calculating modes of collective conformational variability using the AF2 structures alone, however, revealed an interesting local motion involving the putatively catalytic Y291. When the maximum and minimum projections are structurally aligned with the experimental structure, the AF2 models explore conformations where Y291 does flex closer to the C5 and glycosidic oxygen of the ligand (*SI Appendix*, Fig. S10*B*).

Overall, the analyses suggest that a combination of both domain closure and local side-chain movement would allow Y291 to come within at least 4 Å of C5 while the position of H444 has the potential to vary greatly with respect to Y291, and therefore the +1 subsite.

### Proposed Catalytic Mechanism.

Our mutational analysis revealed that three amino acid residues, N238, Y291, and H444, were the key catalytic residues in *Bt*PL33^HA^ (*SI Appendix*, Table S2). The amide of N238 likely acts as a charge stabilizer for the enolate anion transition state and is not predicted to move significantly during transition from the open to closed states. In contrast, Y291 moves within 4 Å from C5 and under 5 Å from the glycosidic oxygen in the closed state but, with further local and global motions, may be even closer in the catalytically competent state. H444 is always >6.5 Å from either C5 or the glycosidic oxygen but does lie within ~3 Å of Y291 in the Apo crystal structures.

**Fig. 7.**
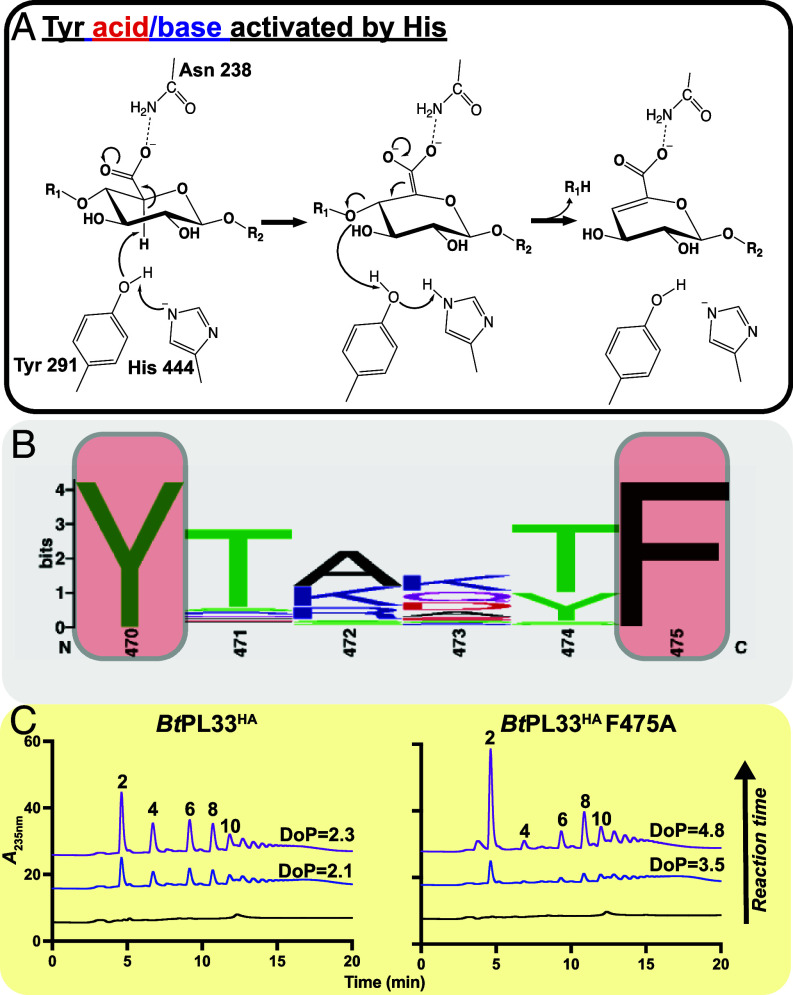
Proposed catalytic mechanism and amino acids affecting processivity. (*A*) The figure proposes an E1cB elimination where proton abstraction precedes proton donation to the leaving group. His444 activates Tyr291 which then abstracts a proton from C5, leading to an anionic transition state stabilized by N238, and as bond cleavage occurs, Y291 donates the abstracted proton to the glycosidic oxygen which becomes the new reducing end (R_1_) of the leaving group. (*B*) Weblogo (a graphical representation of sequence conservation) generated from the 175 sequences used to examine residue conservation showing the high degree of conservation of Y470 and F475. (*C*) High-performance anion exchange chromatograms of wild-type *Bt*PL33HA (5 nM) and its F475A (128 nM) variant at early stages of reaction where <13% of the total disaccharide product had been generated. The numbers 2, 4, 6, 8, and 10 indicate the degree of polymerization (dp) of the HA products. The degree of processivity (DoP) ratio is the amount of dp2 product/amount of dp4, dp6, dp8, dp10 product and is used as a measure for the amount of processivity displayed by *Bt*PL33HA and its variants. Substrate concentrations well above *K*_M_ were deployed and protein concentrations were normalized using *k*_cat_ values. Appropriate time points for each protein were selected by matching the amount of disaccharide produced relative to the wild-type timepoints being used. The same time course was followed for all proteins as were the buffer conditions of 10 mM 2-(N-morpholino)ethanesulfonic acid pH 6.0 with 150 mM NaCl. Graphs are representative examples of duplicate experiments.

We propose that in the closed catalytic state, H444 activates Y291 by abstracting a proton from its phenol hydroxyl, which allows it to act as a base and abstract a proton from C5 leading to formation of the enolate anion transition state which is stabilized by N238. Y291 then donates the abstracted proton from C5 to the glycosidic oxygen and thus O1 of the leaving group sugar. This would be consistent with a *syn* elimination via an asynchronous E1cb mechanism (Fig. 7*A*).

### Loop Residue F475 Controls Processivity.

*Endo*-acting CAZymes can be dissociative or processive in nature. When targeting large polymers, dissociative *endo*-acting enzymes cleave the substrate randomly and have an equal chance of cleaving any bond and will produce large oligosaccharides at the earliest reaction timepoints with few, if any, minimal cleavable units. *Endo*-processive enzymes make an initial random cleavage but, instead of dissociating, move along the glycan change. This has the consequence of producing the minimal cleavable unit at the earliest reaction timepoints alongside the larger oligosaccharides. The greater the proportion of minimal units produced by an *endo*-acting enzyme at these early timepoints, the greater the degree of processivity.

Upon catalysis, *Bt*PL33^HA^ adopts a tunnel topography with the residues Y470 and F475, clamping the substrate against the hydrophobic platform (Fig. 5). This residue pair is present in all PL33 sequences analyzed, and we hypothesized they would be important for processivity, allowing the enzyme to slide along the substrate (Fig. 7*B*). Thus, we mutated Y470 to Ala and Phe, and F475 to Ala and Trp. Interestingly, F475A displayed a significant increase in processivity (Fig. 7*C*, *SI Appendix*, Fig. S11, and Dataset S3), generating a greater proportion of disaccharide minimal units, relative to other products, at the early stages of the reaction, but this was accompanied by a reduced rate.

### The Tunnel Length Determines Substrate Selection.

**Fig. 8.**
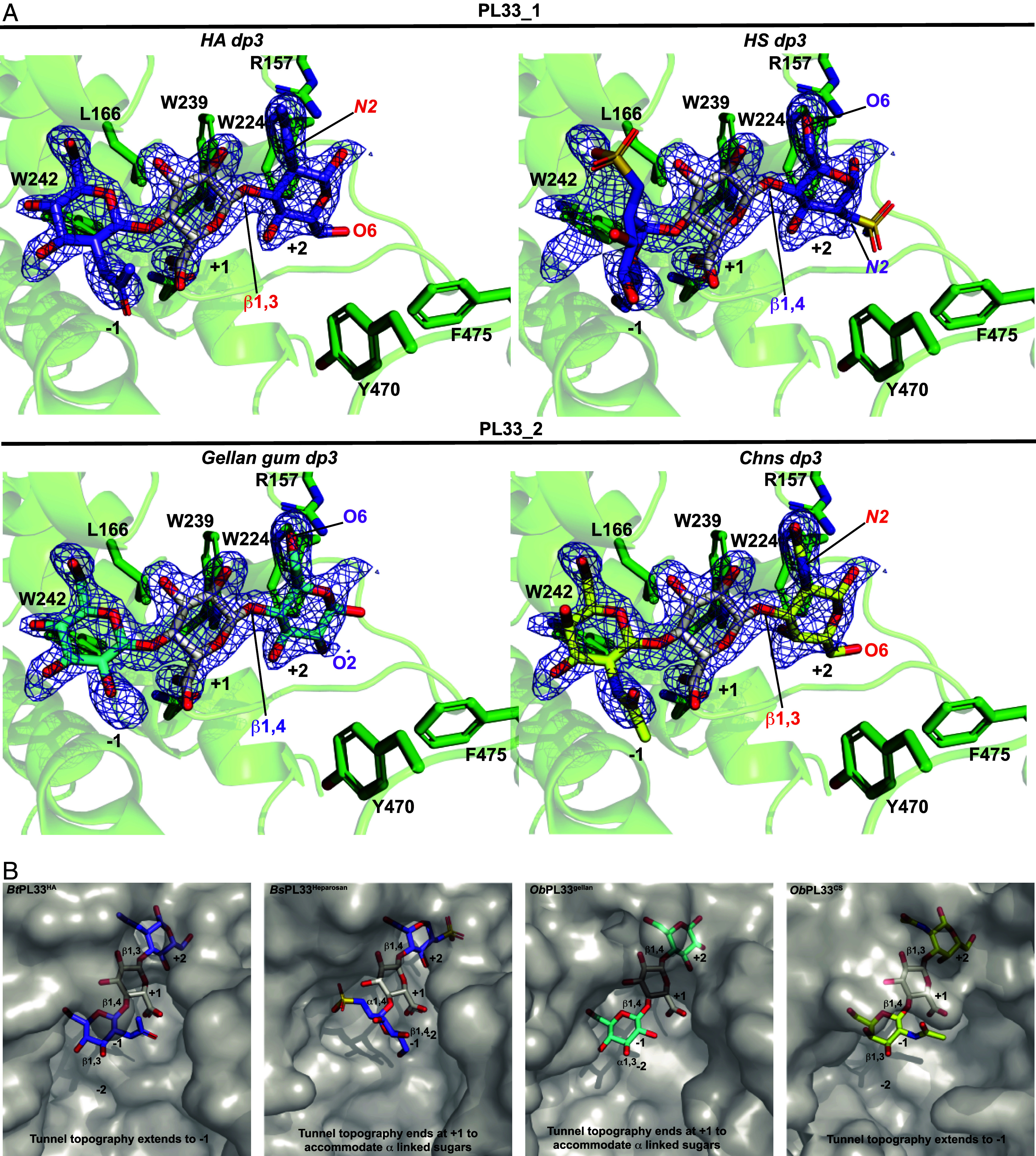
Tunnel topography and length drives glycan specificity. (*A*) Shows the modeling of *Bt*PL33^HA^ with HA, heparosan, gellan gum, and unsulfated chondroitin sulfate (Chns) trisaccharides into the electron density for the modeled a HA trisaccharide (Fig. 4*A*). The electron density for the modeled trisaccharide was used as it showed lower levels of disorder. The trisaccharides were built and regularized in JLigand and fitted in Coot using real space refinement and bond regularization. (*B*) Surface representations of the AF2 models of (*Left* to *Right*) *Bt*PL33^HA^, *Bs*PL33^Heparosan^, *Ob*PL33^gellan^, and *Ob*PL33^CS^ overlaid with the trisaccharide substrates from *A*.

**Fig. 9.**
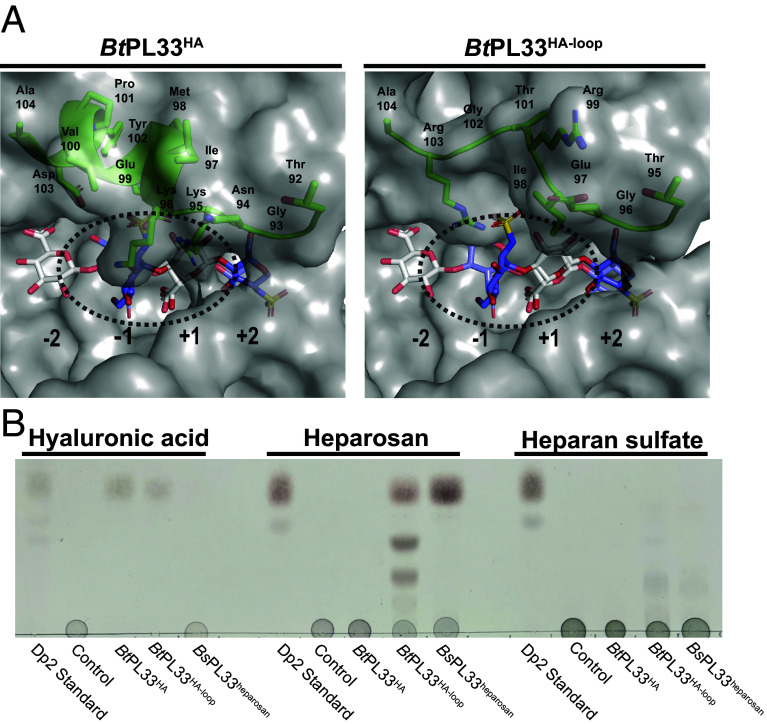
Expanding substrate specificity through tunnel design. (*A*) AF2 models of *Bt*PL33^HA^ and *Bt*PL33^HA-loop^ modeled with both a HA tetrasaccharide and a HS trisaccharide. (*B*) Thin layer chromatography showing an end point activity assay of *Bt*PL33^HA^, *Bt*PL33^HA-loop^, *Bs*PL33^heparosan^ against HA, heparosan, and HS. Enzyme concentrations of 5 µM and substrate concentrations of 5 mg/mL (2.5 mg/mL HA) were deployed and ran overnight for ~16 h at 37 °C. Buffer conditions were 5 mM 3-(*N*-Morpholino)propanesulfonic acid pH 7.0 with 75 mM NaCl.

The +2/+1 subsite of PL33 provides little substrate discrimination. The +2 subsite forms an “aromatic clamp” and can accommodate the sugar rings of D-GlcNAc, D-GalNAc, and D-glucose (which can be β1,3 or β1,4 linked) by “flipping” the +2 sugar (Fig. 8*A*). The +1 subsite of PL33 family members is highly conserved and specific for the binding of GlcA (Fig. 8*A*) while processivity is conferred by the composition of the tunnel architecture that forms upon domain closure. Substrate specificity between PL33 enzymes must therefore be driven by the negative subsites. We detected subfamily variance at the base of the −1 subsite, which has a Trp in PL33_1 and a Val in PL33_2 (*SI Appendix*, Fig. S5). This is accompanied by a covariant substitution of position 301 (His in *Bt*PL33^HA^) to Tyr which then lies atop the -1 subsite Val (*SI Appendix*, Fig. S12). Although the −1 and −2 subsites appear to make no significant substrate interactions, AF2 models of the closed conformations suggest significant differences in their openness that could drive specificity (Fig. 8*B*). For example, the extended tunnel topography of *Bt*PL33^HA^ and *Ob*PL33^CS^ results in greater occlusion of the −1 sites compared to *Bs*PL33^heparosan^ and *Ob*PL33^gellan^. The openness of the −1 subsite of *Bs*PL33^heparosan^ and *Ob*PL33^gellan^ may therefore enable accommodation of α-linked sugars, which drastically alter polysaccharide chain directions, and this difference in shape would be likely to impede binding to the more occluded −1 sites of *Bt*PL33^HA^ and *Ob*PL33^CS^ (Fig. 8*B*). Of note, *Bt*PL33^HA^ and *Ob*PL33^CS^ target substrates with similar shapes and their tunnel topographies reflect this. Although *Bt*PL33^HA^ exhibits its highest catalytic rate toward HA, it is active against the structurally related CS, while *Ob*PL33^CS^ only displays activity on CS. This indicates that sulfation is an important factor in the enzyme’s substrate preferences. Interestingly, *Po*PL33 displayed weak activity on HA, DS, CS, and heparosan. Analysis of the top five AF2 models suggests that the tunnel topography is more open than *Bt*PL33^HA^ but less open than *Bs*PL33^heparosan^ explaining its broader specificity but weaker activity due to a less substrate-specialized tunnel (*SI Appendix*, Fig. S13). Validation of this hypothesis was achieved through inserting the tunnel-forming loop from *Bs*PL33^heparosan^ into *Bt*PL33^HA^. AF2 models predict a more open tunnel at the −1/+1 subsites of the chimeric enzyme, *Bt*PL33^Hep-loop^ (Fig. 9*A*) which maintained activity on HA but also acquired activity against heparosan and HS (Fig. 9*B*), albeit at a catalytic cost (*SI Appendix*, Table S4).

## Discussion

### The PL33 Family Exhibits GAG Substrate Plasticity.

The PL33 family members as a whole display activity against the structurally distinct GAGs, heparosan, CS, and HA. Most PL families possessing GAG degrading enzymes target either the structurally related heparosan, heparin, and HS with mixed linkages of α1,4-β1,4, or CS, DS, and HA with mixed linkage of β1,4-α/β1,3. For instance, PL12, PL13, PL15, and PL21 GAG PLs have only been shown to target heparin/HS (31–35), while PL6, PL16, PL23, PL29, PL30, and PL35 only target CS/DS/HA (21, 36–38). Most characterized members of PL8 target CS and HA, but one member has been shown to target heparin/HS. Thus, the plasticity for structurally distinct GAGs is uncommon and, in the PL33 family, is facilitated by the promiscuity of the +2 subsite and the openness of the −1/−2 subsites.

### Advantages of Processivity against HA.

Although it is encoded at a distal locus, *Bt*PL33^HA^ is upregulated with PUL^CS/DS/HA^ which is primarily responsible for the catabolism of CS, DS, and HA. Owing to the greater structural complexity of CS and DS, their metabolism is contingent on the cooperative activities of multiple exo- and endo-acting enzymes (20). Catabolism of the much simpler HA, however, only requires *Bt*PL33^HA^ due to the *endo-*acting processive nature of this enzyme, simultaneously generating both new reducing/nonreducing ends and limit disaccharide products, thereby obviating the need for dissociative *endo-* and *exo*-acting enzyme pairs. However, a balance must be achieved between processivity and dissociation for the process to remain efficient. Processive hydrolysis of HA is a viable strategy owing to its simple, homogenous structure, whereas the variable sulfation patterns and sugar composition of CS and DS make efficient processive action more challenging.

### An Evolutionary Conserved Fold, Catalytic Apparatus, and Mode of Action.

**Fig. 10.**
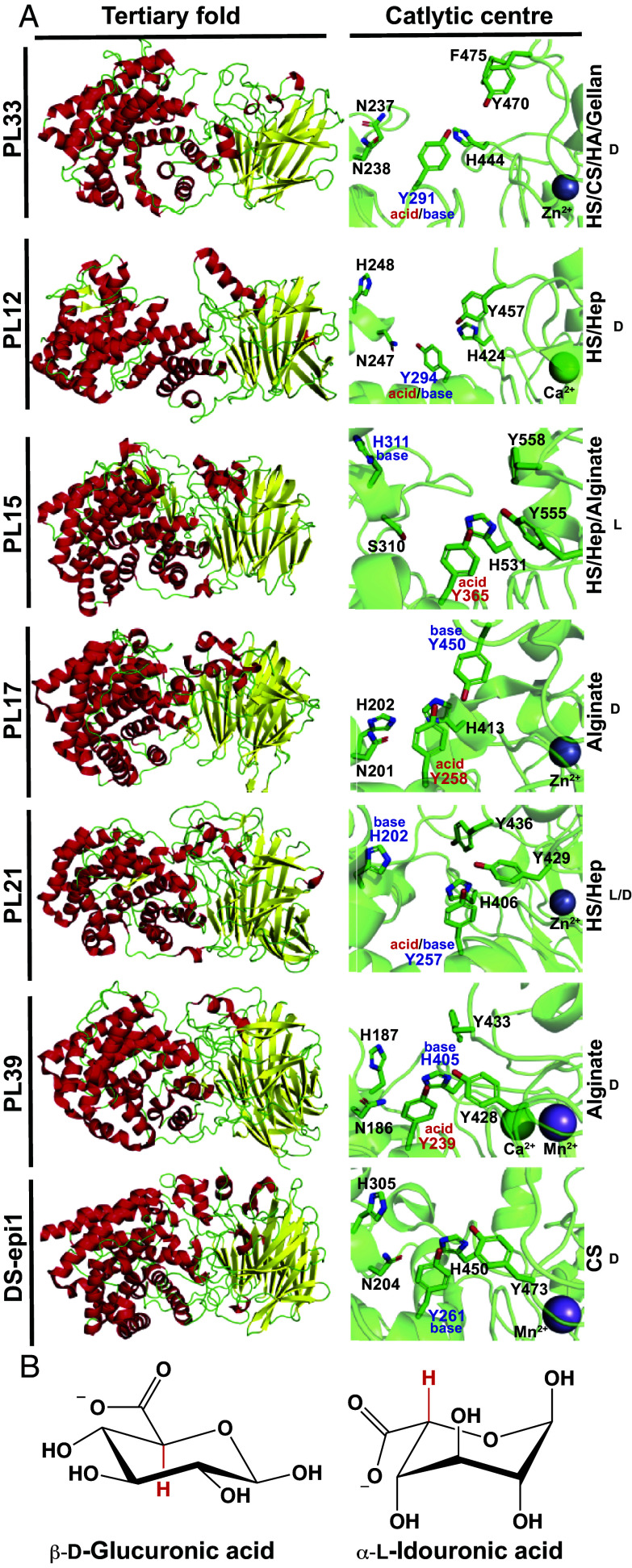
PL33 has an evolutionarily conserved fold and catalytic apparatus. (*A*) (*Left*) Comparison of the tertiary structure of several lyase families and the homo sapien epimerase Ds-epi1 demonstrating the conservation of the two domain (α/α)_6_ + antiparallel β sheet fold. (*Right*) Close-up view of the key catalytic residues, a Tyr291+His444 dyad topped by a second Tyr470 (*Bt*PL33^HA^ numbering), with more variable residues that, although conserved within the family, are not conserved across the enzyme families depicted. In general, the residue position in the sequence is well matched. The substrate targeted is written on the right with the literature-established preference for L or D-uronic acids. (*B*) The structure of D-glucuronic acid and L-idouronic acid targeted by the enzyme families in (*A*).

Comparison of PL33 with other PL families reveals that PL12, PL15, PL17, PL21, and PL39 are all structurally related, possessing an (α/α)_n_ + antiparallel β sheet fold. PL12 (31), PL21 (35), and PL33 target GAGs, while PL17 (39) and PL39 (40) target the marine glycan alginate, and PL15 targets both substrates (34, 41). These substrates both contain both L- and D-uronic acid sugars. The families share most of the catalytic apparatus identified in PL33 in a similar spatial configuration (Fig. 10*A*), having rmsd values of ~3 to 4 Å (Dataset S4). However, despite their conservation, the proposed catalytic roles of these residues differ dramatically depending on whether the +1 sugar (uronic acid) has D- or L-configuration (Fig. 10*A*).

Supported by structural and kinetic data from the literature, PL12 (31, 42), PL21 (32), and PL33 all utilize the orthologous residues Y291/Y294/Y257 as an acid/base when D-GlcA is in the +1 subsite. In contrast, against L-IdoA, PL15 (41) and PL21 (32) utilize the PL33 Y291 orthologs, Y365 and Y257, as catalytic acids and the residues H311 and H202, as catalytic bases. Finally, PL17 (39) and PL39 (40), targeting D-mannuronate in alginate, also utilize the PL33 Y291 ortholog, Y258 and Y259, as a catalytic acid, but utilize Y450 and H405, respectively, as the catalytic base, orthologous to Y470 and H444 in PL33. It would appear that the +1 catalytic apparatus shared by these PL families provides plasticity to act on D- and L-uronic acids, which orient their C5 proton ~180° relative to one another (Fig. 10*B*); orientations that allow proton abstraction from both configurations. Furthermore, these families have been shown to display processivity (40, 43), to exhibit conformational shifts (31, 41) (*SI Appendix*, Fig. S14), and to house a structural metal ion within the β sheet domain (31, 34, 35, 39, 40).

All of these structural and mechanistic features have also been shown for the human DS epimerase DS-epi1 (44–46), which is proposed to utilize Y261 (orthologous to the acid/base Y291 in PL33) as a catalytic base (44). The catalytic acid in DS-epi1, that donates a proton to C5 to invert its stereochemical configuration, remains unidentified but, may be inferred by comparing the catalytic apparatus of lyases that target both GlcA and L-IdoA and share mechanistic and structural similarity to DS-epi1. It is clear that there is an evolutionary relationship between DS-epi1 and the lyases described here, but its exact nature is unclear. Low sequence identity between the enzyme families generally limits the value of phylogenetic analyses. Furthermore, although a divergent evolutionary path with a common ancestor seems likely considering structural, catalytic, and mechanistic similarities, a convergent path cannot be ruled out.

PL33 is a PL family targeting structurally diverse GAGs, as well as gellan gum. Its members contain two domains, and closure of these domains around the substrate is necessary for catalysis. This creates a tunnel topography at the positive subsites that drives an *endo*-processive mode of action and defines substrate specificity. The fold, mode of action, and catalytic apparatus found in PL33 are all conserved across several other PL families, as well as among eukaryotic GAG epimerases, indicating a related evolutionary origin.

## Methods

### Recombinant Protein Production.

Genes were amplified by PCR using the appropriate primers and the amplified DNA cloned in pET28a using NcoI/XhoI or NheI/XhoI restriction sites generating constructs with either N- or C-terminal His_6_ tags. Recombinant genes were expressed in *E. coli* strains BL21 (DE3) or TUNER (Novagen), containing the appropriate recombinant plasmid, and cultured to mid-exponential phase in LB supplemented with 50 μg/mL kanamycin at 37 °C and 180 rpm. Cells were then cooled to 16 °C, and recombinant gene expression was induced by the addition of 0.1 mM isopropyl β-D-1-thiogalactopyranoside; cells were cultured for another 16 h at 16 °C and 180 rpm. The cells were then centrifuged at 5,000× g and resuspended in 20 mM (4-(2-hydroxyethyl)-1-piperazineethanesulfonic acid) (HEPES), pH 7.4, with 500 mM NaCl before being sonicated on ice. Recombinant protein was then purified by immobilized metal ion affinity chromatography using a cobalt-based matrix (Talon, Clontech) and, after a wash with resuspension buffer, eluted with a step gradient of 10, 50, and 100 mM imidazole in resuspension buffer. Proteins were then analyzed by sodium dodecyl sulfate polyacrylamide gel electrophoresis (SDS-PAGE) gel for purity and appropriately pure fractions dialyzed into 10 mM HEPES pH 7.0 with 150 mM NaCl. If proteins were being carried forward for structural experiments, they were concentrated in centrifugal concentrators with a molecular mass cutoff of 30 kDa, after SDS-PAGE analysis, and loaded onto a 16/60 S200 superdex size exclusion column. Fractions from these purifications were then subject SDS-PAGE analysis and fractions judged to >95% pure pooled and concentrated in centrifugal concentrators with a molecular mass cutoff of 30 kDa for further downstream structural analyses. Protein concentrations were determined by measuring absorbance at 280 nm using the molar extinction coefficient calculated by ProtParam on the ExPasy server (web.expasy.org/protparam/).

### Activity Screening.

Polysaccharide substrates were prepared as 0.4% solutions in 100 mM Tris HCl pH7.8 and 50 mM NaCl except for gellan which was prepared in 100 mM Tris HCl pH7.8 without NaCl to avoid gelation. To identify PL33 substrates, 200 μL reactions containing 0.2% substrate and suitably diluted enzyme (0.5 to 5 μM per assay) were incubated for various time lengths (up to 16 h) at 25 °C under shaking. The reaction was terminated by boiling for 10 min. Reaction mixtures were then filtered on 0.2 mm PES membranes and the filtrates were analyzed by assaying reducing sugars using the ferricyanide assay as described before (21) or absorbance at 235 nm. Analysis of degradation products was performed by gel permeation chromatography using a Superdex peptide 10/300 (GE Healthcare) column connected to a high-performance liquid chromatography Ultimate 3000 system (Thermo Fisher). Injection volume was 50 μL and the elution was performed at 0.5 mL min^−1^ in 0.1 M NaCl. Oligosaccharides were detected by differential refractometry (Iota 2 differential refractive index detector, Precision Instruments). Degradation products were analyzed by NMR following the protocol described before (21).

## Supplementary Material

Appendix 01 (PDF)

Dataset S01 (XLSX)

Dataset S02 (XLSX)

Dataset S03 (XLSX)

Dataset S04 (XLSX)

## Data Availability

The crystal structure dataset generated has been deposited in the Protein Data Bank (PDB) under the following accession numbers: 8R75 (47), 8R6Z (48), 8R70 (49), 8R71 (50), 8R72 (51), and 8R73 (52). Information on all other data and materials is contained within the main manuscript and supplemental information.
