# Supplementary material for: Bacterial polysaccharide lyase family 33: Specificity from an evolutionarily conserved binding tunnel
Source: Proc Natl Acad Sci U S A. 2025 Feb 11;122(7):e2421623122. doi: 10.1073/pnas.2421623122 (PMC11848413; doi:10.1073/pnas.2421623122)
Supplement: Supplementary file 1 — Appendix 01 (PDF) [file pnas.2421623122.sapp.pdf]

PNAS

Supporting Information for

Bacterial polysaccharide lyases employ a conformationally induced mode of catalysis which is shared with mammalian epimerases

Mélanie Loiodice[1], Elodie Drula[2,3], Zak McIver[4], Svetlana Antonyuk[5], Arnaud Baslé[6], Marcelo Lima[7], Edwin A Yates[5], Dominic P Byrne[5], Andrew Leech[8], Shahram Mesdaghi[5,9], Daniel J Rigden[5], Sophie Drouillard[1], William Helbert[1], Bernard Henrissat[10,11], Nicolas Terrapon[12,13], Gareth SA Wright[14], Marie Couturier[1]*, Alan Cartmell[4,15,16]*

[1]Univ. Grenoble Alpes, CNRS, CERMAV, 38000, Grenoble, France.
[2]Architecture et Fonction des Macromolécules Biologiques (AFMB), CNRS, Aix-Marseille Université, Marseille, France.
[3]INRAE, UMR 1163, Biodiversité et Biotechnologie Fongiques, Marseille, France.
[4]Department of Biology, University of York, Heslington, York, YO10 5DD, UK
[5]Department of Biochemistry, cell and systems biology, Institute of systems, molecular and integrative biology, University of Liverpool, Liverpool L69 7ZB, U.K.
[6]Newcastle University Biosciences Institute, Medical School, Newcastle University, Newcastle upon Tyne NE2 4HH, UK.
[7]School of life sciences, Huxley Building, Keele University, Keele, Staffordshire, ST5 5BG
[8]Technology Facility, Department of Biology, University of York, Heslington, York, YO10 5DD, UK
[9]Computational Biology Facility, MerseyBio, University of Liverpool, Crown Street, Liverpool L69 7ZB, U.K
[10]Department of Biological Sciences, King Abdulaziz University, Jeddah 23218, Saudi Arabia
[11]Department of Biotechnology and Biomedicine (DTU Bioengineering), Technical University of Denmark, DK-2800 Kgs. Lyngby, Denmark
[12]Architecture et Fonction des Macromolécules Biologiques, CNRS, Aix-Marseille University, F-13288 Marseille, France.
[13]USC1408 Architecture et Fonction des Macromolécules Biologiques, Institut National de la Recherche Agronomique, F-13288 Marseille, France.
[14]School of life sciences, University of Essex, Wivenhoe Park, Colchester, CO4 3SQ, U.K
[15]York Structural Biology Laboratory (YSBL), University of York, Wentworth Way, York, YO10 5DD, U.K.
[16]York Biomedical Research Institute (YBRI), University of York, Wentworth Way, York, YO10 5DD, U.K.

*To whom correspondence should be addressed:
marie.couturier@cermav.cnrs.fr
alan.cartmell@york.ac.uk

This PDF file includes:

Supporting text

Figures S1 to S14

Tables S1 to S8

Legends for Datasets S1 to S4

SI References

Supplemental methods

X-ray crystallography experiments

After purification, proteins were carried forward in the same eluent as used for the size exclusion chromatography; 10 mM HEPES pH 7.5, 150 mM NaCl. All proteins were then concentrated in centrifugal concentrators with a molecular mass cutoff of 30 kDa. Sparse matrix screens were set up in 96-well sitting drop TTP Labtech plates (400-nL drops) using an SPT mosquito crystallisation robot, or in sitting drop intelli plates (400-nL drops) using an Arts Robbins gryphon robot. Initial *Bt*PL33^{HA} apo crystals were obtained at 20 mg/mL in 20% PEG 3350 and 200 mM Na/K tartrate and 20 % PEG 6000, MES pH6.0, and 100 mM Ammonium chloride. For *Bt*PL33^{HA} Substrate bound *Bt*PL33$^{HA-Y291A}$ crystals were obtained at 20 mg mL^{-1} in 20 % PEG 6000, MES pH6.0, and 100 mM Ammonium chloride and were soaked overnight with 1 mM of a HA tetrasaccharide prior to fishing; crystals were cryo-cooled with the addition of 20% PEG 400. For initial hits of *Bc*PL33^{HA} apo crystals were grown in 10 % PEG 8000,0.1 M HEPES pH 7.5 with 8 % ethylene glycol: crystals were cryo-cooled with the addition of ethylene glycol up to 25%. Data were collected at Diamond Light Source (Oxford) on beamlines I24 and I04-1 (0.98, 1.22, and 1.33 Å) at 100 K. The data were integrated with XDS[1] and scaled and merged with Aimless[2,3]. Five percent of observations were randomly selected for the R_{free} set. The phase problem was solved by molecular replacement using the program Phaser or Molrep with a model generated through the RoseTTAfold server. Models then underwent recursive cycles of model building in Coot[4] and refinement cycles in Refmac5[5]. The models were validated using Coot[4] and MolProbity[6]. Carbohydrates were made using Jigand[7]. Structural Figures were made using Pymol (The PyMOL Molecular graphics system, Version 2.0 Schrodinger, LLC.) and all other programs used were from the CCP4 suite[8]. The data processing and refinement statistics are reported in **Table S6 and S7**.

Site-Directed Mutagenesis.

Site-directed mutagenesis was conducted using a modified PCR-based QuikChange protocol. Appropriate primer pairs with the mutation were designed to have overlapping 5' regions of 18 bps and 12 bps non-overlapping at the 3' end (**Table S5**). These primers, 0.3 μM were mixed with 10 ng of target plasmid and subject to 18 cycles of 95°C for 30s, 50°C for 30s, and 72°C for 4 min; an initial single cycle of 95°C was done preceding the 18 cycles. CloneAmp HiFi PCR Premix (Takara Bio) was utilised for all PCR reactions. A 0.8 % agarose gel was ran to visualise successful reactions and to those Dpn1 was added prior to transforming 2-5 μl in 100 μl of Top10 supercompetent cells. These were then plated onto kanamycin containing LB-agar plates and grown overnight at 37°C. Colonies were then picked, grown up in LB, and plasmid DNA prepped and sequenced to confirm the correct sequence.

Spectrophotometric based polysaccharide lyase assays

Polysaccharide lyase kinetics were performed using a Biochrom Libra S22 UV/Vis Spectrophotometer equipped with an 8 position water heated cell changer

connected to a peltier water circulator set to 25°C. Reactions were carried out in black walled quartz cuvettes with a 500 μl volume and monitored at a wavelength of 235 nm. Reactions were ran for 10 minutes prior to the addition of enzyme to ensure a zero baseline was acquired. Enzyme concentrations from 5 nM to 5 μM were deployed but at the higher protein concentrations interference from the peptide bond signal started to become evident. *Streptococcus equi* HA (100 kDa) was used for all enzyme kinetics except those for the mutants Y470A, Y470F, F475A, and F475W where *Streptococcus equi* HA (70-130 kDa) was used. Substrate hydrolysis was limited to <10 % to maintain linearity and substrate concentrations straddling the K_M were deployed to calculate individual K_M and k_{cat} values by fitting the data using non-linear regression and the equation $V_0 = V_{max}[S]/[K_M]+[S]$. Where K_M exceeded the maximum substrate deployed k_{cat}/K_M values were calculated by linear regression using the equation $V_0 = (V_{max}/K_m)[S]$. Substrate concentrations were halved and doubled to assess linearity of the reaction rates to ensure substrate concentrations were significantly $<K_M$. GraphPad Prism 10 software was used to analyse all kinetic data.

Differential scanning fluorimetry

Thermal shift/stability assays (TSAs) were performed using a StepOnePlus Real-Time PCR machine (LifeTechnologies) and SYPRO-Orange dye, at a 1:1000 dilution, (emission maximum 570 nm, Invitrogen) with thermal ramping between 20 and 95°C in 0.3°C step intervals per data point to induce denaturation of purified, folded, *Bt*PL33^{HA} and its various mutant variants. The melting temperature (Tm) corresponding to the midpoint for the protein unfolding transition was calculated by fitting the sigmoidal melt curve to the Boltzmann equation using GraphPad Prism, with R^2 values of ≥0.99, as described in[9]. Data points after the fluorescence intensity maximum were excluded from the fitting. Changes in the unfolding transition temperature compared with the control curve (ΔT_m) were calculated for each ligand. A positive ΔT_m value indicates that the ligand stabilises the protein from thermal denaturation, and confirms binding to the protein. All TSA experiments were conducted using a final protein concentration of 5 µM in 100 mM Bis-Tris-Propane (BTP), pH 7.0, and 150 mM NaCl, supplemented with the appropriate ligand concentration. Three independent assays were performed for each protein and protein ligand combination.

Thin layer chromatography (TLC)

End point assays were analysed by TLC by spotting 2 µL of sample onto silica plates and resolved in butanol:acetic acid:water (2:1:1) running buffer. The plates were dried, and the sugars were visualized using diphenylamine stain (1 ml of 37.5% HCl, 2 ml of aniline, 10 ml of 85% H_3PO_3, 100 ml of ethyl acetate and 2 g diphenylamine) and heated at 450°C for 2-5 min with a heat gun. Protein buffer conditions were 100 mM MES pH6.0 with 150 mM NaCl and reactions ran at 25 °C using 5 μM of protein. HA substrate concentration of 2.5 mg/ml was used (*Streptococcus equi* HA 70 – 100 kDa).

High performance anion exchange chromatography (HPAEC)

Product profiles were analysed by HPAEC using an ICS-6000 thermofisher (Dionex) system coupled to a single channel variable wavelength detector (VWD) set to a wavelength of 235 nm. A PA-200 Carbopac analytical column (3 x 250 mm), with a preceding PA-200 guard column (3 x 50 mm) was used to separate enzyme produced products. The eluents used were Ultrapure H_2O (Eluent A), and Ultrapure H_2O with 3 M NaCl. For the sample separation a gradient of 15 % eluent B was ran 0-30 minutes, then switched to 100 % eluent B for 30-40 mins, then ran back into eluent A for 40-50 minutes. All samples and standards were diluted 1:100 into 300 μl and 100 μl of this loaded onto the column. Protein buffer conditions for the product profile assays were 100 mM MES pH6.0 with 150 mM NaCl and reactions ran at 25 °C. Protein concentrations deployed were as follows: *Bt*PL33HA (5 nM) and its variants Y470A (280 nM), Y470F (1 μM), F475A (128 nM) and F475W (2.24 nM), with a HA substrate concentration of 2.5 mg/ml (*Streptococcus equi* HA 70 – 100 kDa and HA 100 kDa).

Phylogenetic and sequence analyses

Protein accessions of non-fragmentary PL33 proteins were extracted from the CAZy database[10] in July 2022, and used to retrieve the corresponding amino-acid sequences from the NCBI database. To have a taxonomically homogeneous dataset, we split the 462 sequences into phylum-level sub-sets on which we performed a CD-HIT[11] clustering at an identity threshold of 90%. The final set is composed of Actinobacteria (32 proteins), Bacteroidetes (56 proteins), Firmicutes (43 proteins), Proteobacteria (24 proteins; mostly Alphaproteobacteria) and diverses other phyla (one or two proteins). We finally included in this dataset our ten targets, resulting in 175 full-length sequences for which we extracted the PL33 catalytic domain. The domain sequences were aligned using MAFFT (version 7.453[12]) using the accuracy-oriented method with the maxiterate option set to a value of 1000. TrimAl v1.2[13] was applied to remove poorly aligned regions from then alignment with the automated option. The alignment was used to compute a distance matrix based on maximum likelihood distances[14]. The resulting distance matrix was used as input to reconstruct a phylogenetic tree with PhyML[15]. The tree rendering, to highlight taxonomical groups and targets studied here, was realized thanks to iTOL webserver[16].

For sequence alignment analysis the PL33 family the sequences from the above phylogenetic analysis were ran on the online MAFFT[12] online server using the default settings and alignments viewed in Jalview[17]. Residue conservation was visualised using the weblogo server[18].

Size exclusion coupled light scattering determination of molecular weight

Molecular masses were determined using an Agilent Multi-Detector System calibrated with bovine serum albumin. Proteins were separated by size exclusion chromatography using an Agilent BioSEC Advance 300 Å, 4.6 x 300 mm column equilibrated with 20 mM tris(hydroxymethyl)aminomethane-HCl pH 7.4, 150 mM NaCl buffer. Light scattering data was collected at 90° and refractive index used to calculate absolute molecular mass.

SAXS data collection and analysis

SAXS data for all proteins was collected at Diamond Light Source on beamline B21 using a size exclusion chromatography step at room temperature. Blank frames were taken prior to protein elution for buffer subtraction purposes. Individual frames recorded over a single chromatography run were averaged based on Rg values and overall similarity on a correlation map in ScÅtter. Two separate chromatography runs were performed and averaged to give the final scattering profile. Data averaging, Guinier approximation and distance distribution function analysis was performed Primus[19]. Molecular envelopes derived from experimental SAXS data were created using DAMMIN without imposition of symmetry restraints. At least 45 structures were averaged using Damaver and aligned with crystal structures or Alphafold2 (AF2) models using Cifsup[19]. Models of BT4410 monomers generated by molecular dynamics simulation were sampled at 1000 time points throughout the 1μs simulation. Each monomer was combined with all other monomers by aligning to residues 83-98, 128-169, 187-220 of the *Bt*PL33^{HA} crystal structure or the corresponding region of *Bc*PL33^{HA} to generate 5×10^5 dimer structures. These were compared against experimental SAXS data using stand-alone FoXS[20] and models ranked according to χ^2 value. AF2 monomers were assembled into dimers as above for *Bt*PL33^{HA} or using the analgous region of *Bc*PL33^{HA}.

Computational sampling of *Bt*PL33 conformational space

All modelling and molecular dynamics simulations were carried out using an Ubuntu 18.04.6 workstation with an AMD Ryzen Threadripper2990WX 32 Core CPU (3.0 GHz) with 64GB RAM, GPU acceleration was performed by an ASUS TUF GeForceRTX 3080 OC LHR 12GB GDDR6X Ray-Tracing Graphics Card, 8960 Core, 1815MHz Boost. *Bt*PL33^{HA} was modelled with AF2 using the ColabFold 1.5.2[21] environment and was executed with or without the 'use_dropout' option which, when enabled, introduces randomness into the modelling. Template search was disabled. MSAs were obtained using the MMSeqs2[22] server, with varied MSA depths; the subsampling was carried out randomly by AlphaFold2[23], and depth values were controlled by modifying 'max_msa_clusters' and 'max_extra_msa' parameters. 1993 *Bt*PL33^{HA} models were constructed; these were filtered to remove misfolded models by removing structures that had a Cα rmsd of more than 1.5Å with respect to the experimental structure thus leaving 1035 models. The experimental structure underwent a 1μs full atom GROMACS[24] molecular dynamics simulation using the CHARMM36[25] force field and from the stable part of the trajectory, a set of 5000 snapshots was obtained. Utilising the experimental structure, a further 1000 models were constructed using CONCOORD[26]. All of the models, from AF2, CONCOORD and GROMACS, were concatenated, and a set of eigenvectors and eigenvalues were calculated with GROMACS *covar* on Cα atoms only. The top three eigenvalues were used as conformational descriptors. A similar analysis was done on the AF2 set alone. The Cα-only maximum and minimum projections were converted to all-atom representations using Pulchra in order to subject them to DynDom analysis.

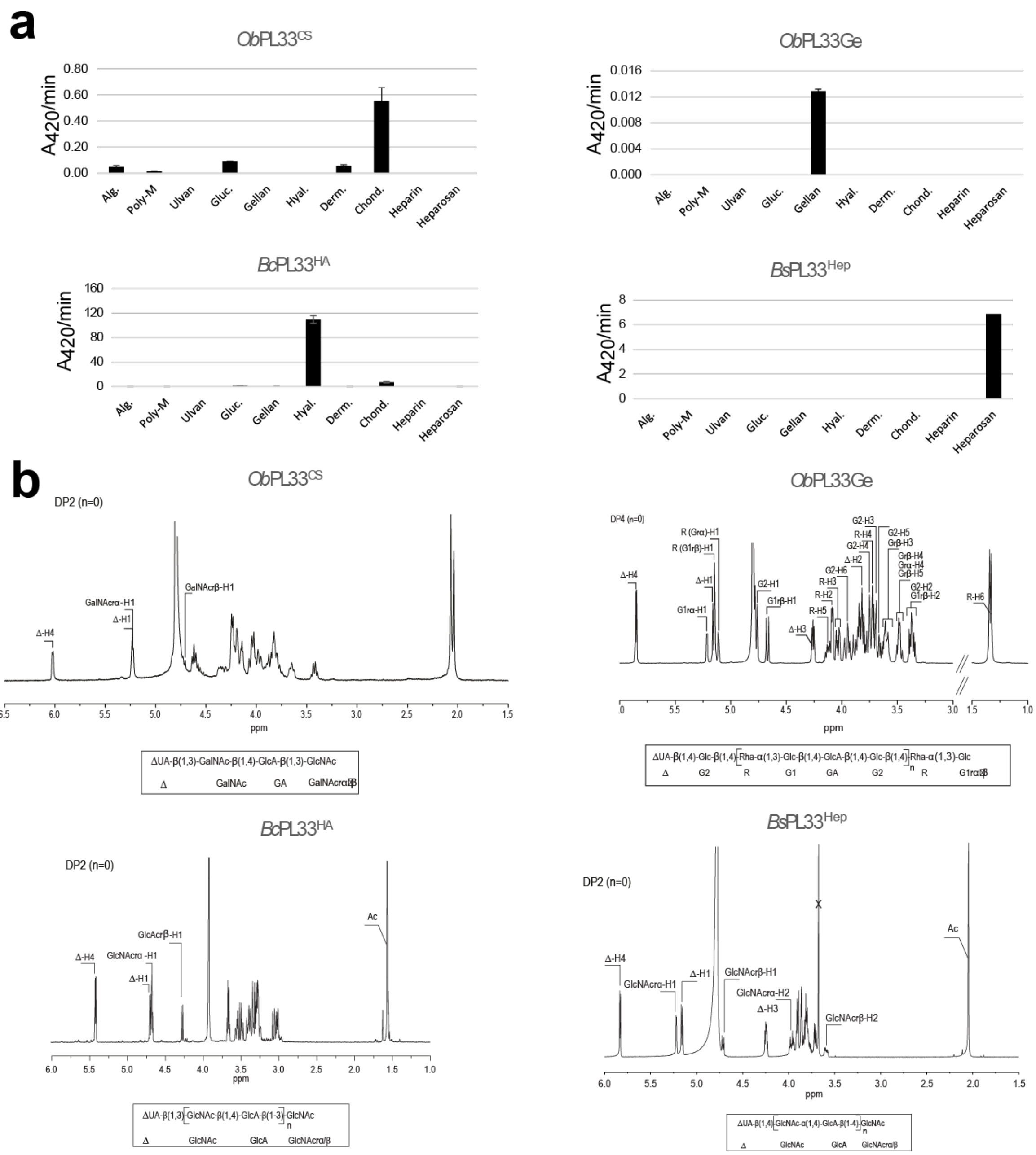

Figure S1. Substrate specificity of PL33 enzymes and analysis of degradation products.

a. Initial velocity of the degradation of uronic acid-containing polysaccharides by PL33 members. Alg : alginate, poly-M: mannuronan, gluc: glucuronan, Hyal: hyaluronic acid, derm: dermatan, chond: chondroitin sulfate. **b.** H NMR spectra of the end-products of each polysaccharide degraded by PL33 lyases. The proton signals were attributed.

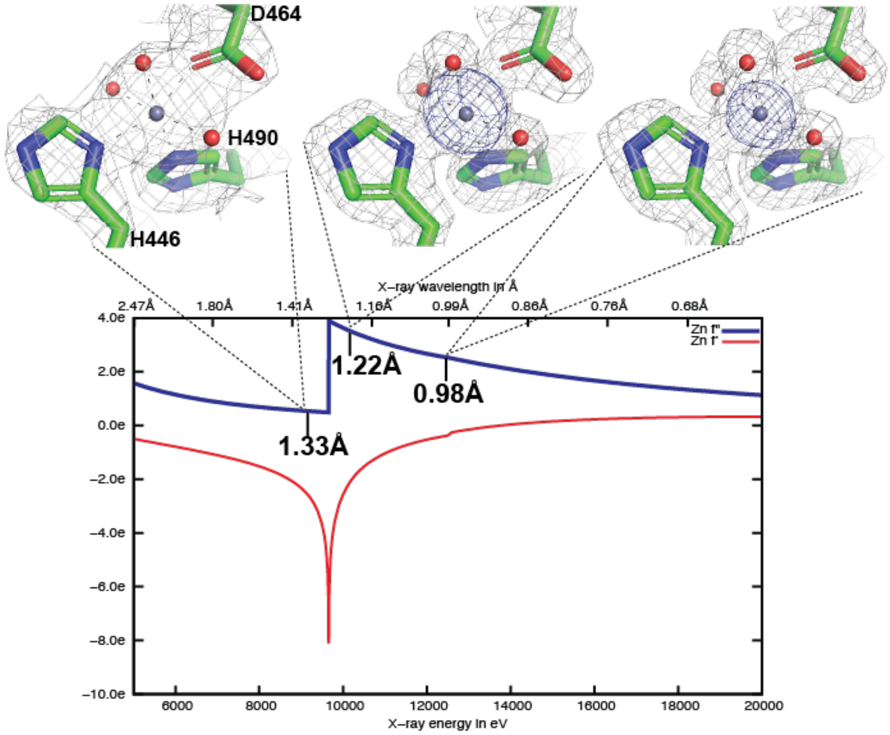

Figure S2. Anomalous scattering experiments for metal identification.
Grey mesh is a weighted 2mF_{obs}-DF_c electron density map, contoured to 1.5σ. From left to right the maps have resolutions of 2.58, 1.92, and 1.8 Å and were collected at wavelengths corresponding to 1.33, 1.22, and 0.98 Å, respectively. In blue mesh is the weighted anomalous difference maps contoured to 5.8 σ. There is an increase of anomalous signal from 0.98 to 1.22 Å but the anomalous signal is absent after the Zinc edge at 1.33 Å.

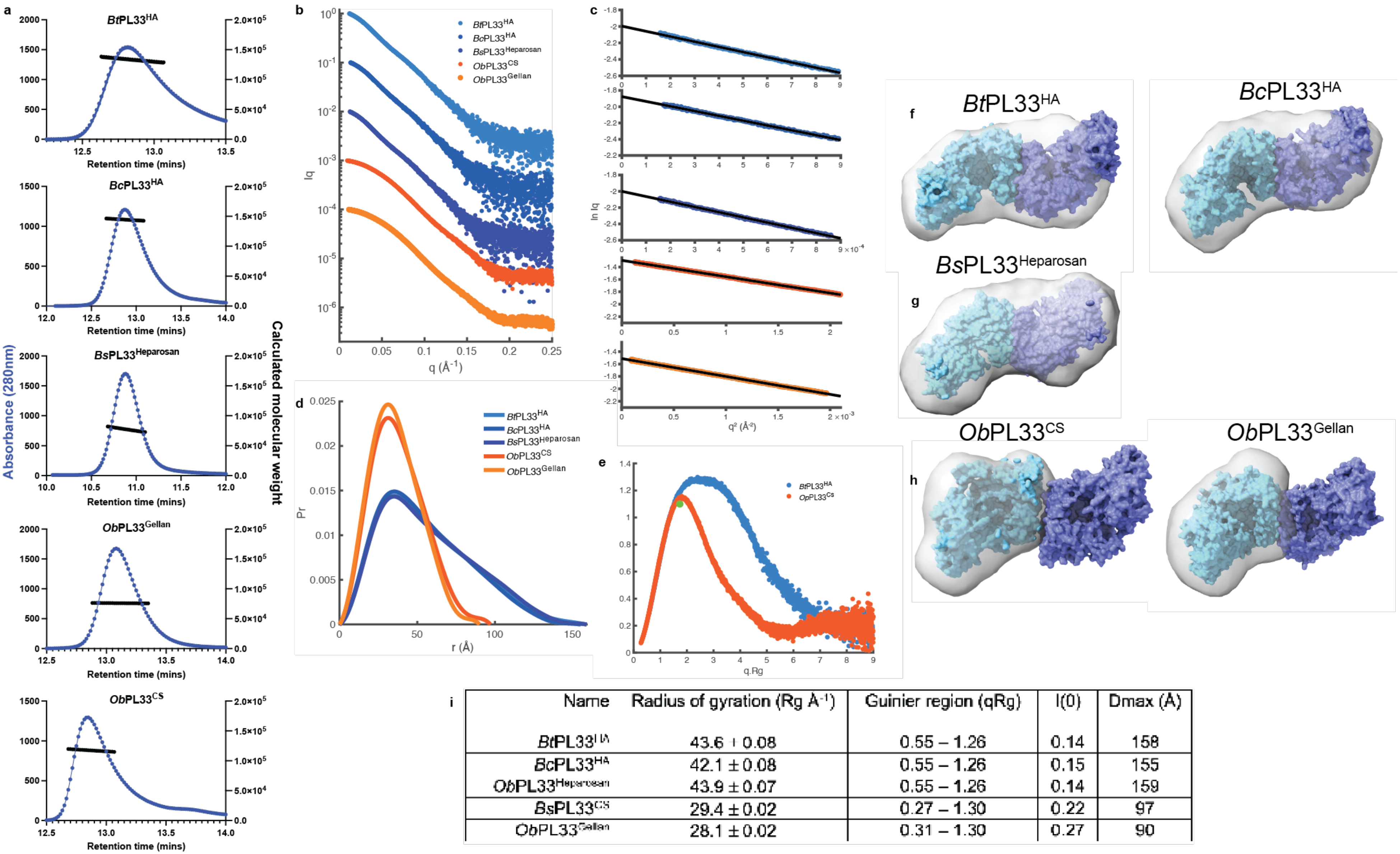

i

Name	Radius of gyration (Rg Å^{-1})	Guinier region (qRg)	I(0)	Dmax (Å)
*Bt*PL33^{HA}	43.6 ± 0.08	0.55 – 1.26	0.14	158
*Bc*PL33^{HA}	42.1 ± 0.08	0.55 – 1.26	0.15	155
*Ob*PL33^{Heparosan}	43.9 ± 0.07	0.55 – 1.26	0.14	159
*Bs*PL33^{CS}	29.4 ± 0.02	0.27 – 1.30	0.22	97
*Ob*PL33^{Gellan}	28.1 ± 0.02	0.31 – 1.30	0.27	90

Figure S3. Quaternary structure and oligomeric states.

a. Size exclusion chromatography-coupled light scattering; **b.** Experimental SAXS scattering data. **c**. Guinier plots of scattering presented in b. **d.** SAXS distance distributions showing larger intra-particle maximum dimension for *Bt*PL33^{HA}, *Bc*PL33^{HA} and *Bs*PL33^{Heparosan} in comparison with *Ob*PL33^{CS} and *Op*PL33^{Gellan}. **e**. Exemplar *Bt*PL33^{HA} and *Ob*PL33^{CS} SAXS data formatted as dimensionless Kratky plots. **f.** Comparison of dimeric *Bt*PL33^{HA} and *Bc*PL33^{HA} crystallographic structures with SAXS-derived molecular envelopes. **g**. Comparison of a dimeric model of *Bs*PL33^{Heparosa}, created using AF2 in homodimer mode, with a SAXS-derived molecular envelope. **h.** *Ob*PL33^{CS} and *Ob*PL33^{Gellan} dimers created by modelling AF2 generated monomers to the *Bt*PL33^{HA} homodimer interface. **i**. Comparison of SAXS parameters for all PL33 described above. In summary, *Bt*PL33^{HA}, *Bc*PL33^{HA} and *Bs*PL33^{Heparosan} form homodimers while *Ob*PL33^{CS} and *Ob*PL33^{Gellan} are monomeric in solution.

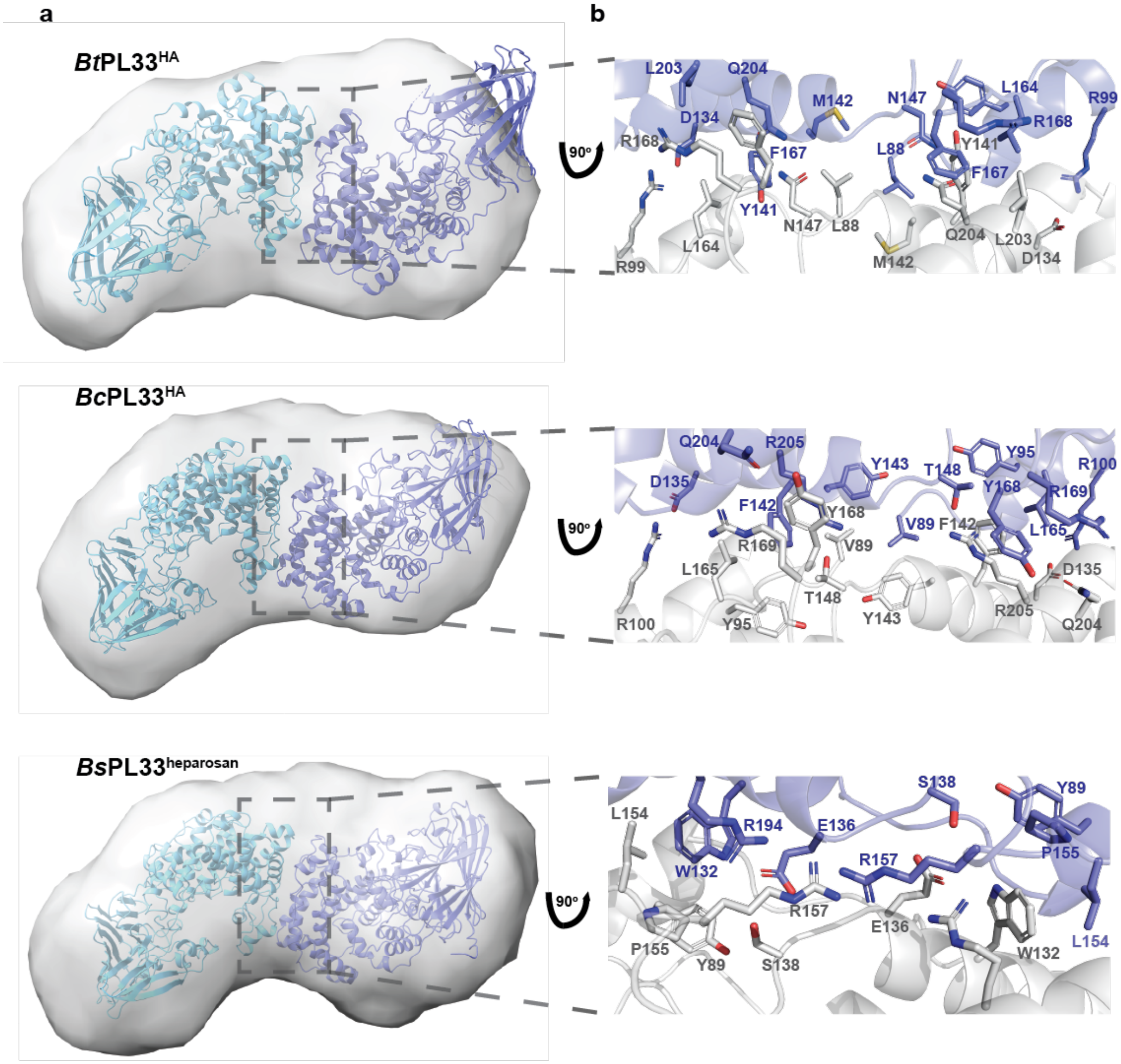

Figure S4. Dimer interfaces for dimeric PL33 enzymes.
The top and middle panel are the crystallographic structure of *Bt*PL33^{HA}, and *Bc*PL33^{HA}, whilst *Bs*PL33^{heparosan} is an AF2 predicted dimer: **a**. Modelling of the dimer into *ab initio* envelopes; **b.** Close-up representation of the dimer interfaces interactions.

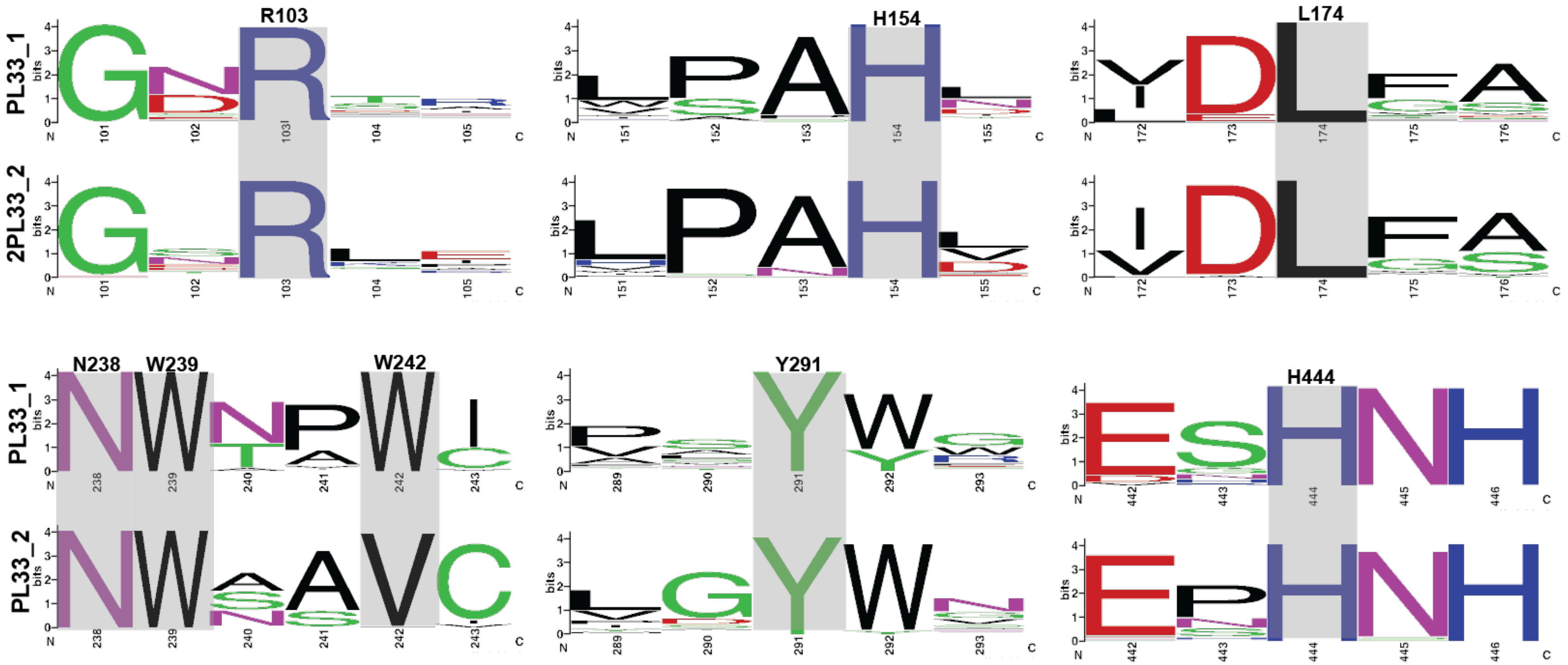

Figure S5. Sequence conservation of key residues.
Weblogo representation of key residues for substrate binding and catalysis.

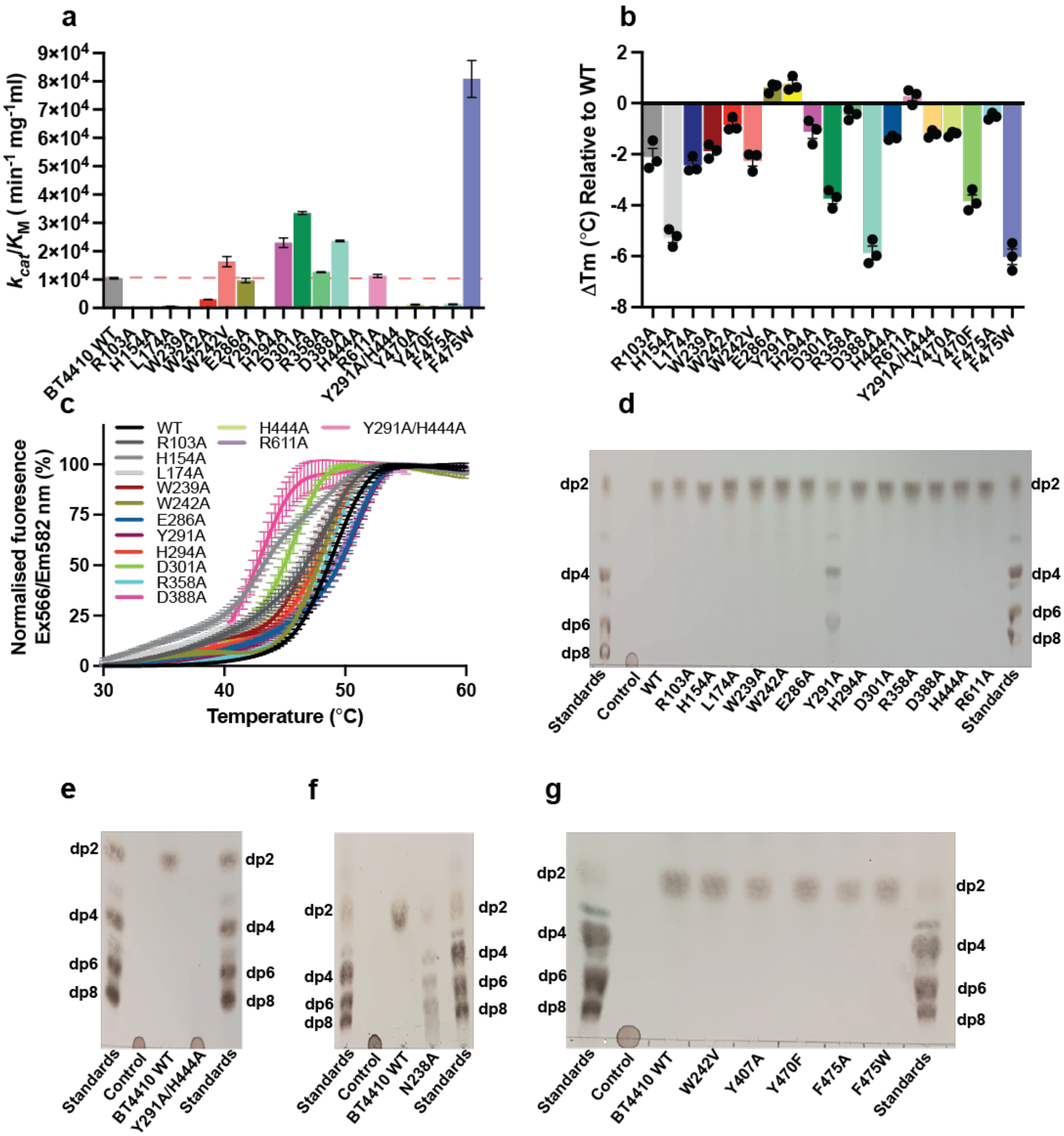

Figure S6. Kinetic and biophysical properties of BT4410^{PL33} and its mutants.
a, Bar chart visualising k_{cat}/K_M values for BT4410^{PL33} and mutants on HA; **b,** ΔT_m values determined using differential scanning fluorimetry (DSF) for BT4410PL33 mutants relative to wildtype; **c,** Thin layer chromatography analysis of BT4410^{PL33} and mutants using 5 μM enzyme and 2.5 mg/ml HA at 37ºC for 20 h, in 100 mM MES, pH 6.0, and 150 mM NaCl; **d,** Normalised DSF values used to calculate the data in **b**; **e, f.** Thin layer chromatography analysis of BT4410^{PL33} and the mutants Y291A/H444A, and N238A, using 5 μM enzyme and 2.5 mg/ml HA at 37ºC for 20 h, in 100 mM MES, pH 6.0, and 150 mM NaCl; **g**. Thin layer chromatography analysis of BT4410^{PL33} and the loop mutants of Y470 and F475A, as well as the -1 subsite mutant W242V, using 5 μM enzyme and 2.5 mg/ml HA at 37ºC for 20 h, in 100 mM MES, pH 6.0, and 150 mM NaCl

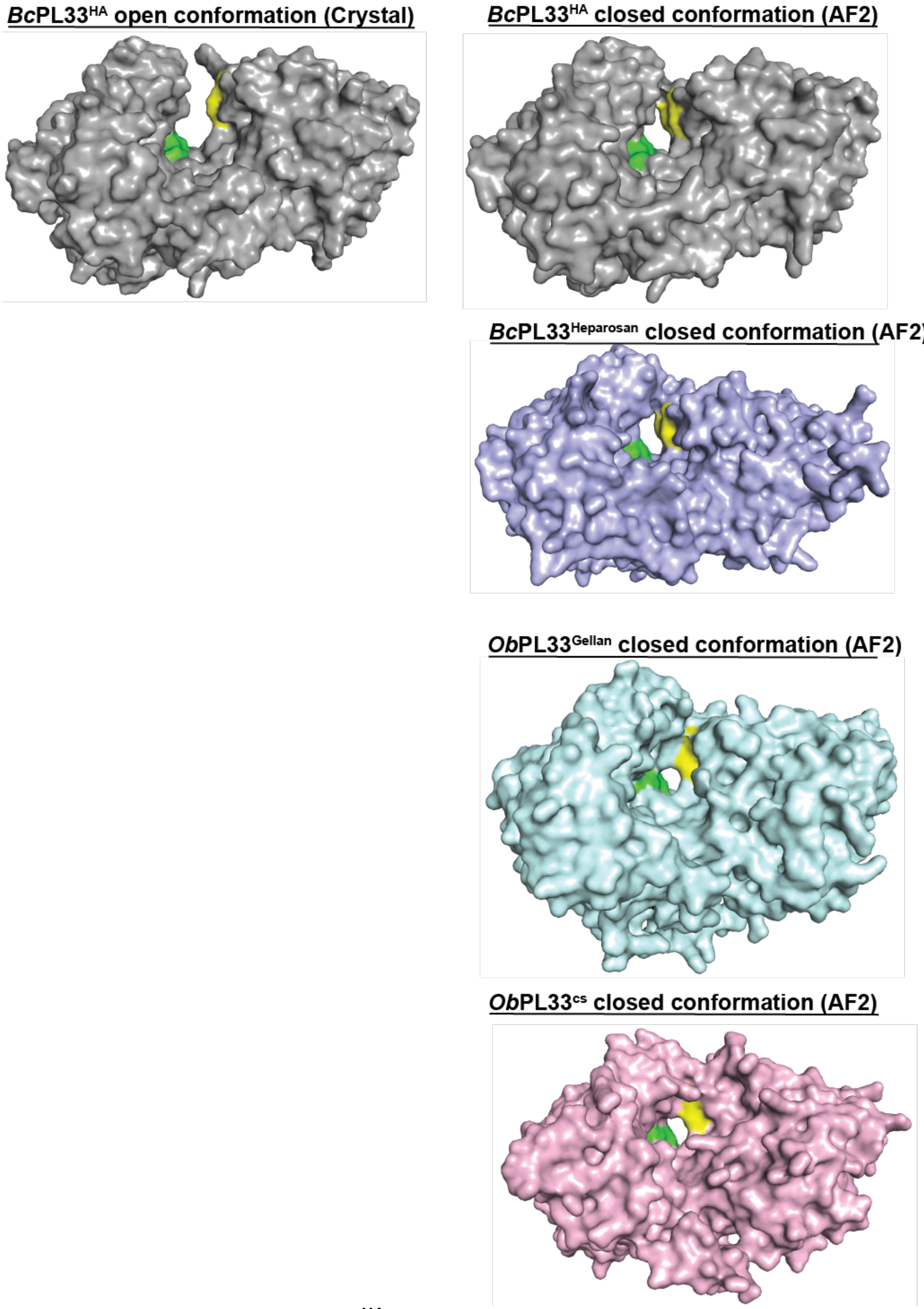

Figure S7. Open state of *Bc*PL33^{HA} crystal structure and closed AF2 models for four characterised PL33 members.

Surface representations of PL33 members in open and closed states. Green represents the orthologous hydrophobic platform of W224/W239/L174/W242 and yellow represents Y470/F475 residues in the loop region which forms the tunnel on closure; numbering from *Bt*PL33^{HA}.

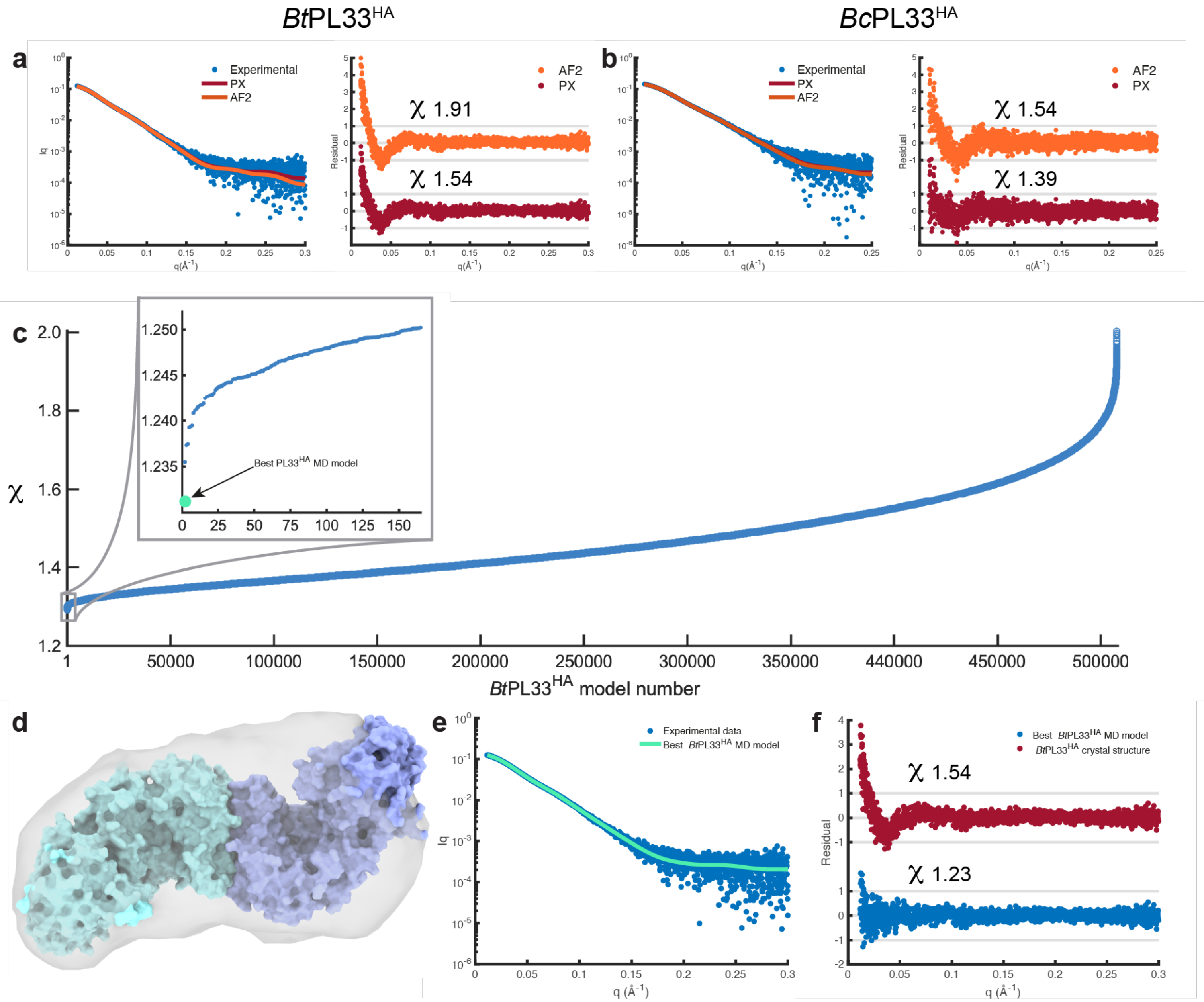

BtPL33HA
BcPL33HA
a
Experimental
PX
AF2
χ 1.91
χ 1.54
b
χ 1.54
χ 1.39
Iq
Residual
q(Å-1)
c
Best PL33HA MD model
χ
BtPL33HA model number
d
e
Experimental data
Best BtPL33HA MD model
q (Å-1)
f
BtPL33HA crystal structure
χ 1.54
χ 1.23

Figure S8. Refining the solution structure of *Bt*PL33^{HA} using molecular dynamics and SAXS.
Comparison of AF2 and crystallographic (PX) models with experimental SAXS data for **a**. *Bt*PL33^{HA} and **b**. *Bc*PL33^{HA}. **c.** *Bt*PL33^{HA} MD models ordered based on chi value against SAXS data. Seven models have chi value less than 1.240. Of these, three dimers share one monomer indicating the modelling process converged on a subset of likely structures all of which are in the open conformation. **d.** *Bt*PL33^{HA} homodimer surface model and SAXS-derived envelope for the best fitting structure. **e.** Comparison of experimental and calculated scattering **f.** Fit residuals for the best fit model and *Bt*PLL33^{HA} crystal structure.

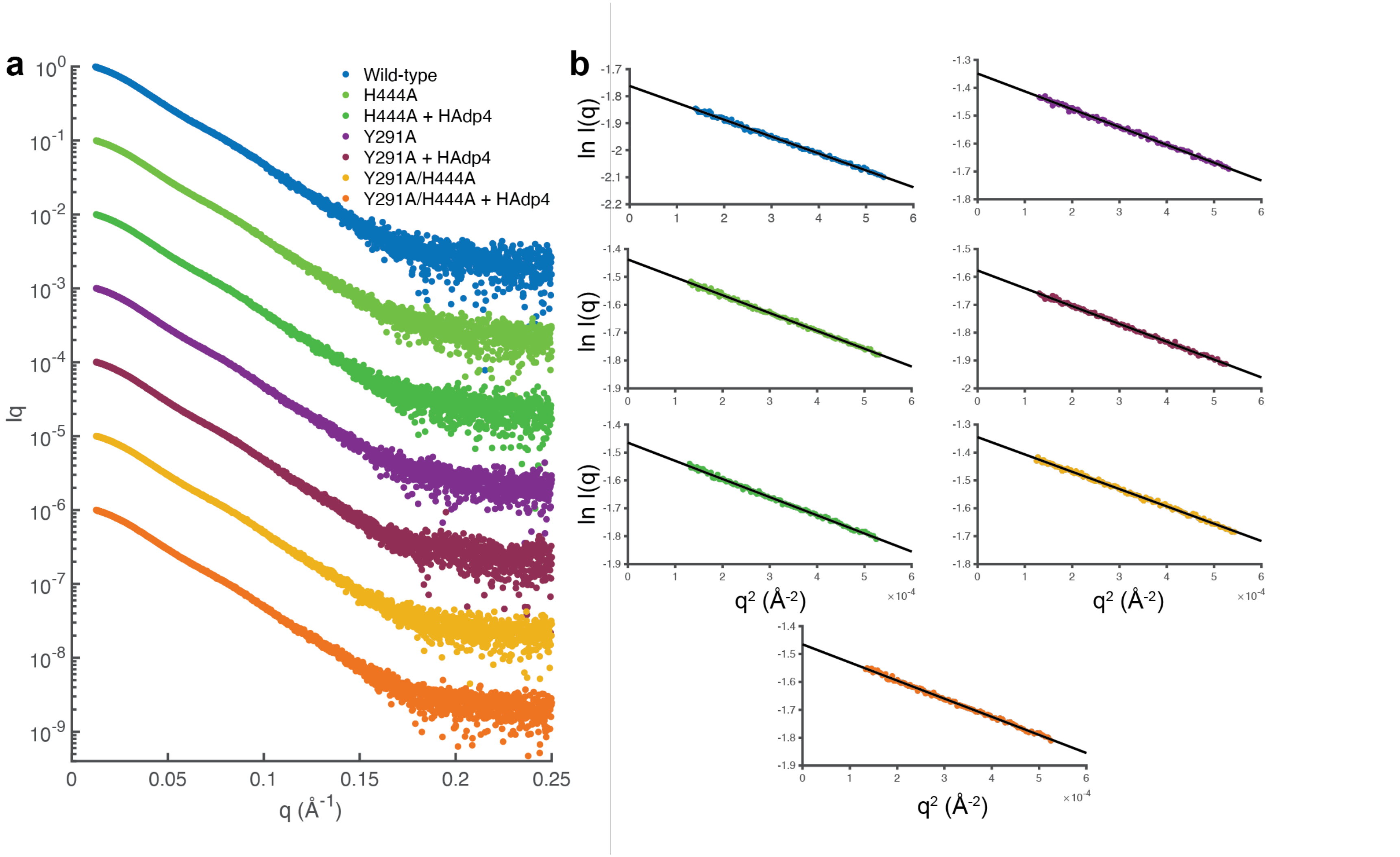

Figure S9. SEC-SAXS data analysis of wildtype and mutant variants with and without substrate.
a. Mutation to the active site with presence or absence of HA dp4 substrate do no affect small angle X-ray scattering by *Bt*PL33^{HA}. **b**. Guinier plots of the data in a.

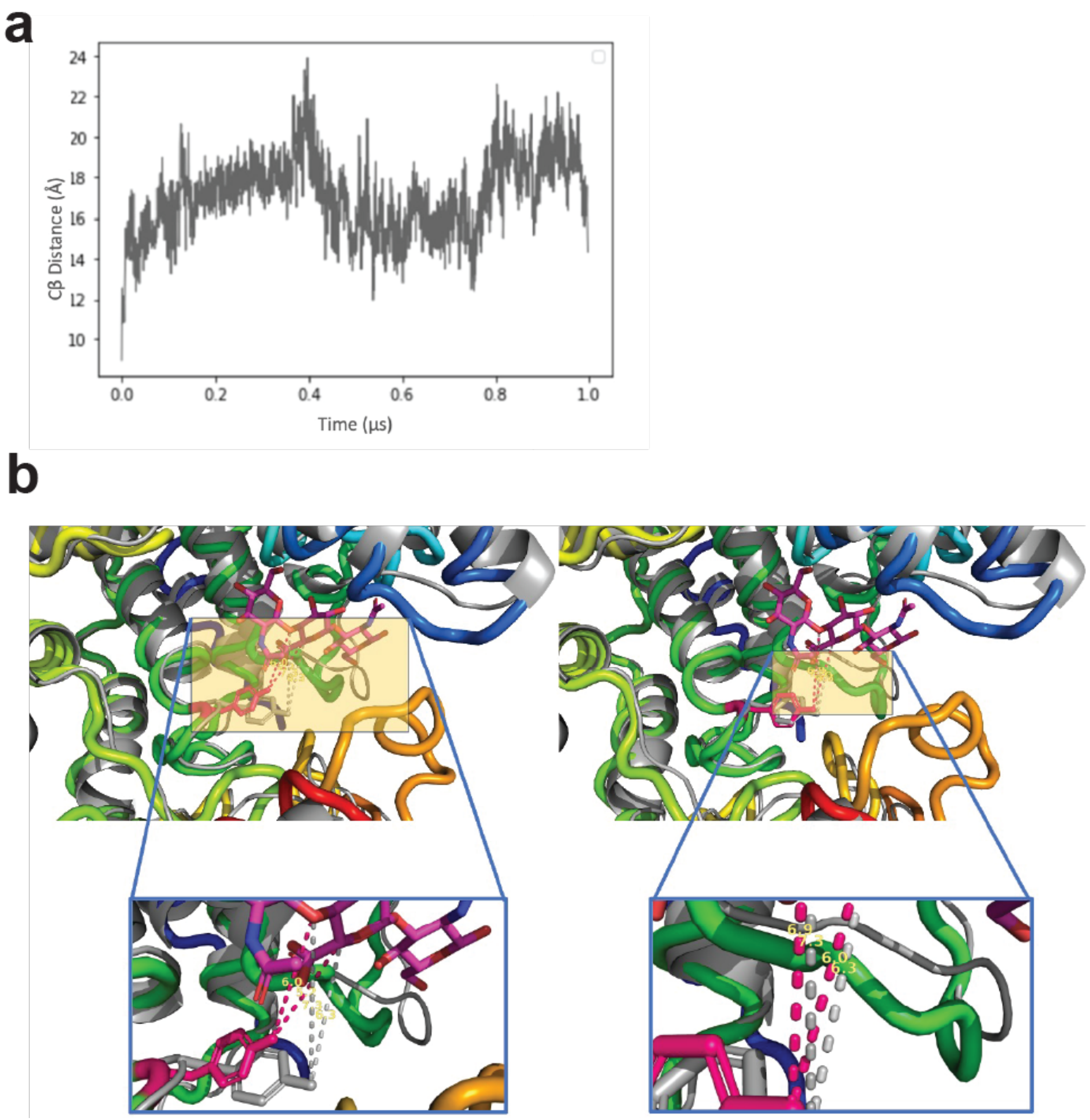

Figure S10. Computational sampling of BtPL33 conformational space.
a. The changes in distance between the Cβ atoms of Y291 (in the $(a/a)_6$ toroid domain) and H444 (in the all-β domain); as the all atom GROMACS simulation progresses these residues move apart. **b.** The maximum and minimum projections along the top eigenvector for the AlphaFold2 generated *Bt*PL33^{HA} models. View shows the catalytic pocket of *Bt*PL33HA; grey is the crystal structure; rainbow (blue, N-terminal to red, C-terminal) is the *Bt*PL33^{HA} maximum (right) and minimum (left) projected model with the catalytic Tyr291 side chain shown as stick and in magenta. Distances (Å) between the tyrosine side chain hydroxyl oxygen and the hyaluronic acid C5 and glycosidic oxygen atoms are highlighted.

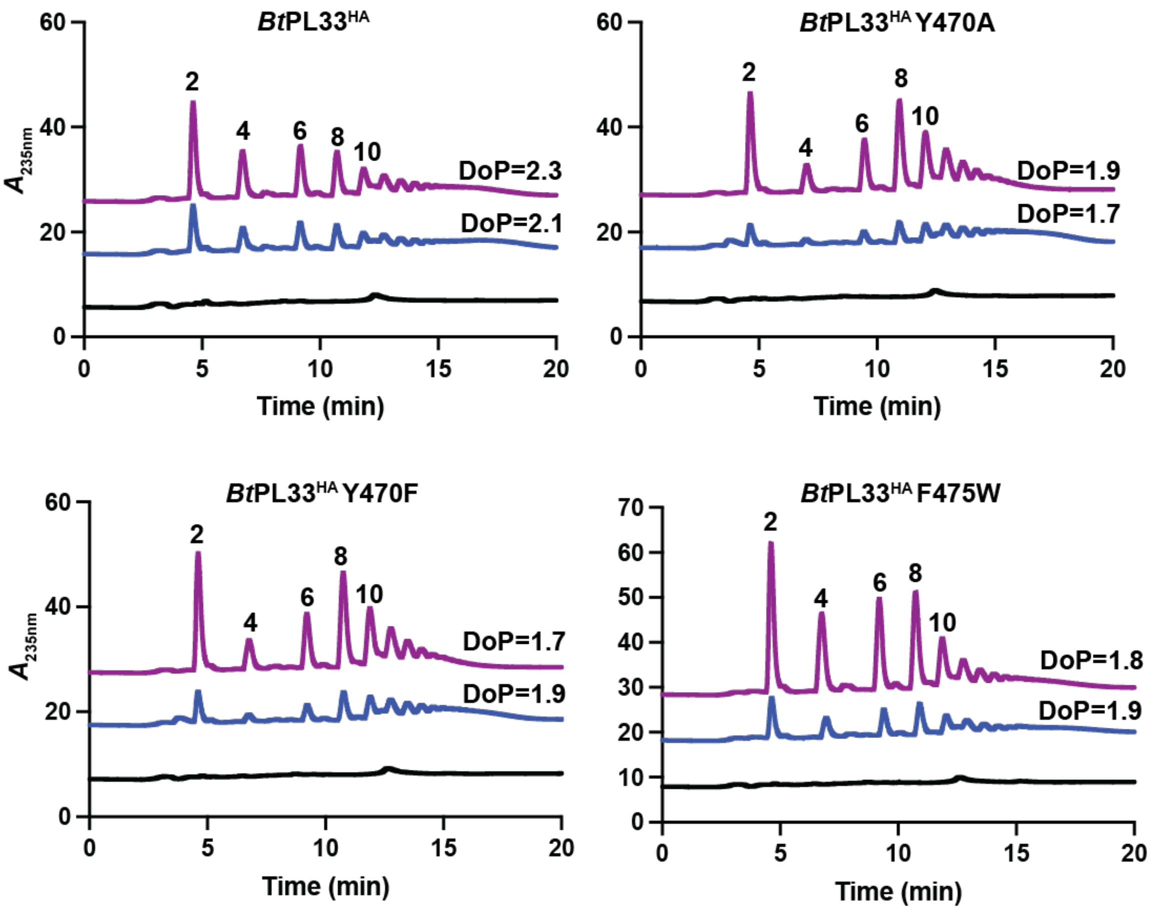

Figure S11. Product profiles of loop mutants.

High performance anion exchange chromatograms of wildtype *Bt*PL33HA and its loop variants at early stages of reaction where <13 % of the total disaccharide product generated. The numbers 2, 4, 6, 8, and 10 indicate the degree of polymerisation (dp) of the HA products. The degree of processivity (DoP) ratio is the amount of dp2 product/amount of dp4, dp6, dp8, dp10 product and used as a measure for the amount of processivity displayed by *Bt*PL33HA (5 nM) and its variants Y470A (280 nM), Y470F (1 μM), and F475W (2.24 nM). Substrate concentrations well above K_M were deployed and protein concentrations normalised using k_{cat} values. Appropriate time points for each protein were selected by matching the amount of disaccharide produced relative to the wildtype timepoints being used. The same time course was followed for all proteins as were the buffer conditions of 10 mM MES pH 6.0 with 150 mM NaCl. Graphs are representative examples of duplicate experiments.

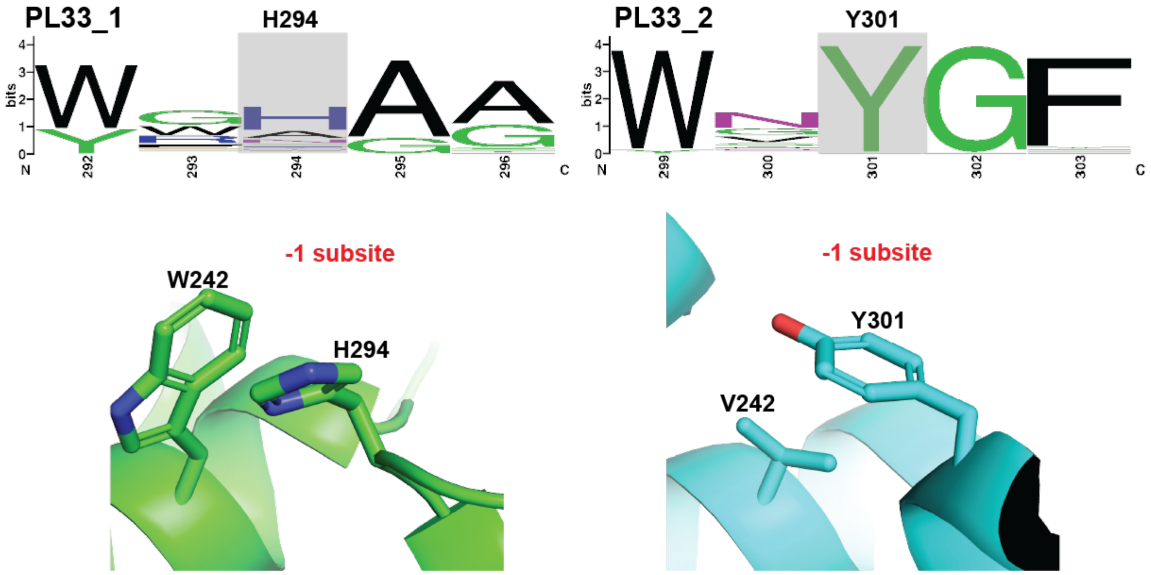

Figure S12. Co-variant mutation at the -1 subsite.
Weblogos and images showing the conservation, and structure, of the of the amino acids at position 301 in response to a switch from W242 in PL33_1 to V242 in PL33_2.

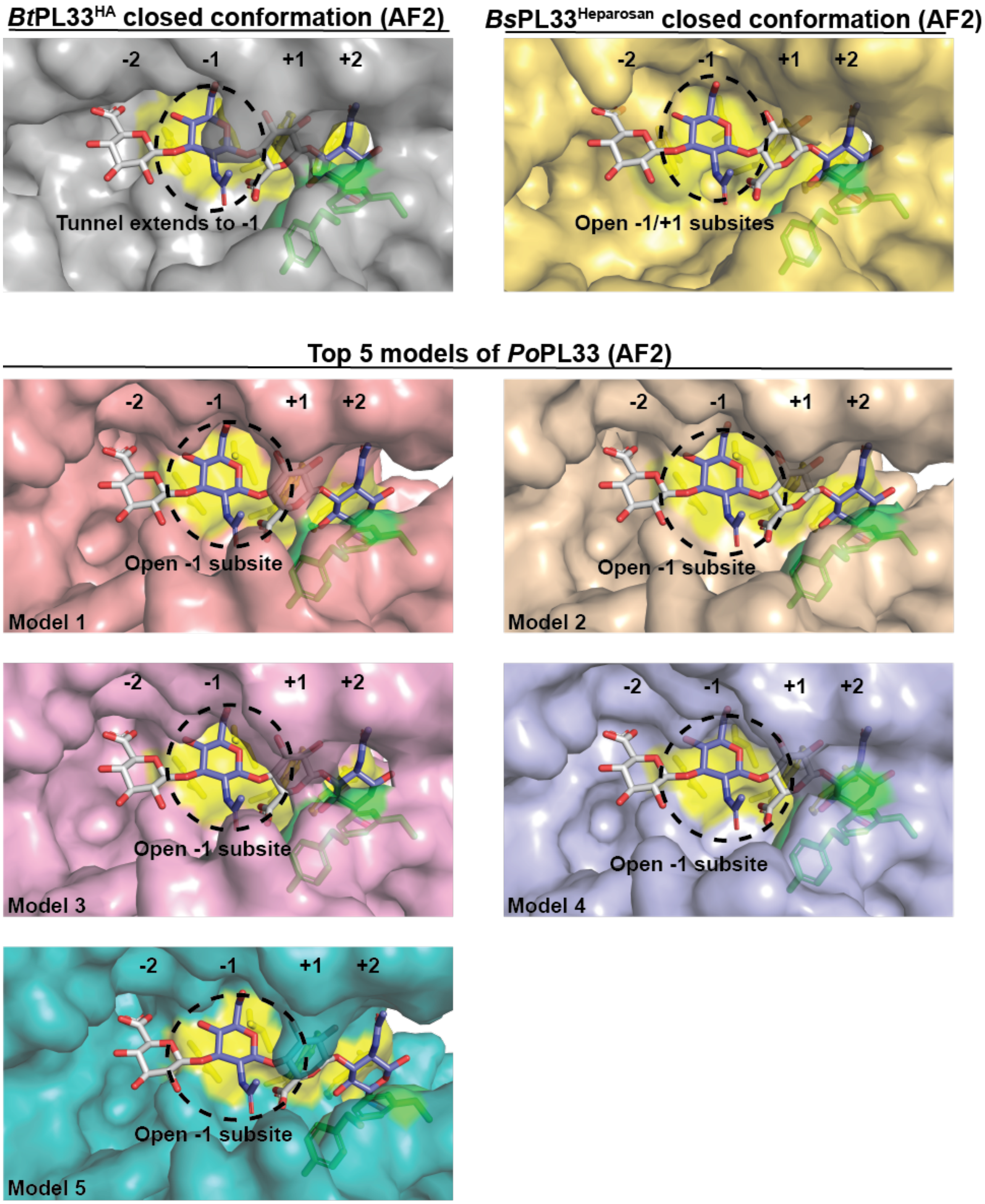

Figure S13. Alphafold2 modelling of the tunnel topography of *Po*PL33 compared to *Bt*PL33^{HA} and *Bs*PL33^{Heparosan}.

The top ranked AF2 models of *Bt*PL33^{HA} and *Bs*PL33^{Heparosan} are shown at the top as the examples of an occluded (*Bt*PL33^{HA}) or an open (*Bs*PL33^{Heparosan}) -1/+1 subsites. Below are the top 5 ranked AF2 models showing the openness of -1/+1 subsites compared to the models *Bt*PL33^{HA} and *Bs*PL33^{Heparosan} above.

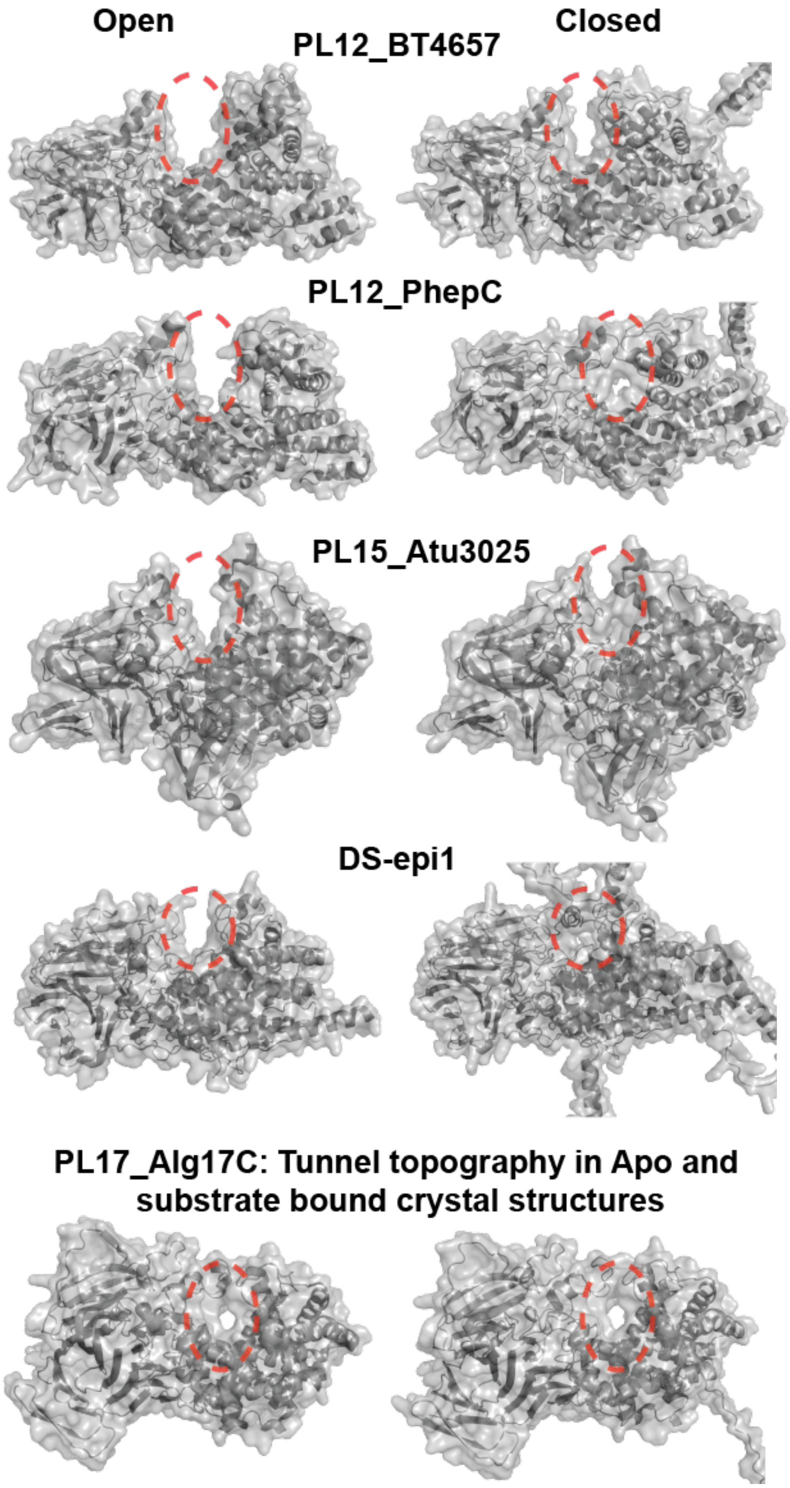

Figure S14. Conformational flexibility by related PL and epimerase families.
The open confirmations are all crystallographic data (PDB codes PL12_BT4657:5MJF, PL12_PhepC:4MMI, PL15_Atu3025:3A0O, DS-epi1:6HZN, PL17_Agl17C:4OK4) whilst the closed confirmations are AF2 models except for PL15_Atu3025 which is a crystallographically-captured closed form (PDB:3AFL). A red circle identifies the area of the catalytic site

Table S1. Substrate preferences of cloned PL33 members

Strains	Gene tag	Short	GenBank	HA	DS	CS	Heparosan	Glc	Gellan
**Subfamily 1**									
Bacteroides cellulosilyticus WH2	BcellWH2_04512	*Bc*PL33^{HA}	ALJ61728.1	+++	-	+	-	-	-
**Bacteroides thetaiotaomicron* VPI-5482	BT4410	*Bt*PL33^{HA}	AAO79515.1	++	+	+	-	NT	NT
Prevotella oris NCTC13071	NCTC13071_00468	*Po*PL33	VEH14491.1	+	+	+	+	-	-
Bacteroides stercoris CC31F	HMPREF1181_01744	*Bs*PL33^{Heparosan}	EPH20528.1	-	-	-	+	-	-
Bacteroides clarus YIT 12056	HMPREF9445_03068	*Bc*PL33^{Hep}	EGF49696.1	-	-	-	-	-	-
**Subfamily 2**									
Maribacter forsetii DSM 18668	P177_DRAFT_02790	*Mf*PL33	WP_209435194.1	-	-	-	-	-	-
Opitutaceae bacterium TAV5	OPIT5_09595	*Ob*PL33^{gellan}	AHF90411.1	-	-	-	-	-	weak
Opitutaceae bacterium TAV5	OPIT5_11155	*Ob*PL33^{CS}	AHF90672.1	-	+	+	-	+	-
Opitutaceae bacterium TAV5	OPIT5_12920	*Ob*PL33	AHF90976.1	-	-	-	-	-	-
Curtobacterium sp. BH-2-1-1	BJK06_00825	*CsPL33*	*AOX64548.1*	-	-	-	-	-	-

+ indicates activity observed on substrate and an increased number of pluses indicates a stronger preference/activity on this substrate. *indicates activity is present but weak – indicates no activity. *data from Ndeh *et al* (https://doi.org/10.1038/s41467-020-14509-4). NT : not tested.

Table S2. Kinetic parameters of *Bt*PL33[HA] and its mutants against hyaluronic acid polysaccharide and oligosaccharides.

Hyaluronic oligosaccharide substrates				
	K_M (mg ml^{-1})	k_{cat} (min$^{-1)}$	k_{cat}/K_M (min^{-1} M^{-1})	Relative to WT k_{cat}/K_M (%)
**HA dp4**	-	-	(7.58 ± 1.85) x 10^6	-
**HA dp6**	-	-	(7.76 ± 2.22) x 10^6	-
**Hyaluronic acid polysaccharide (100 kDa)**				
**Enzyme**	K_M (mg ml^{-1})	k_{cat} (min^{-1})	k_{cat}/K_M (min^{-1} mg^{-1} ml)	Relative to WT k_{cat}/K_M (%)
**WT**	0.23 ± 0.04	(2.30 ± 0.24) x 10^3	(1.05 ± 0.10) x 10^4	100
**R103A**	>0.5	-	(2.48 ± 0.10) x 10^2	2
**H154A**	>0.5	-	(1.71 ± 0.18) x 10^2	1.6
**L174A**	0.44 ± 0.02	(2.40 ± 0.23) x 10^2	(5.53 ± 0.58) x 10^2	5.3
**N238A**	NQ	NQ	NQ	NQ
**W239A**	>0.25	-	(1.01 ± 0.12) x 10^2	1
**W242A**	0.26 ± 0.02	(7.62 ± 0.41) x 10^2	(2.96 ± 0.04) x 10^3	28
**W242V$^+$**	0.09 ± 0.02	(1.59 ± 0.58) x 10^3	(1.63 ± 0.18) x 10^4	155
**E286A**	>0.5	-	(9.66 ± 0.81) x 10^3	92
**Y291A**	NQ	NQ	NQ	NQ
**H294A**	0.10 ± 0.004	(2.39 ± 0.19) x 10^3	(2.30 ± 0.17) x 10^4	219
**D301A**	0.16 ± 0.03	(5.89 ± 0.18) x 10^3	(3.35 ± 0.51) x 10^4	319
**R358A**	0.35 ± 0.10	(4.00 ± 0.45) x 10^3	(1.26 ± 0.20) x 10^4	120
**D388A**	0.13 ± 0.02	(3.05 ± 0.28) x 10^3	(2.36 ± 0.27) x 10^4	226
**H444A**	NQ	*(1.4 ± 0.01) x 10^{-1}	NQ	*6 x 10^{-3}
**Y470A$^+$**	0.04 ± 0.01	(4.10 ± 0.45) x 10^1	(1.14 ± 0.16) x 10^3	11
**Y470F$^+$**	0.05 ± 0.01	(1.20 ± 0.12) x 10^1	(2.95 ± 0.67) x 10^2	2.8
**F475A$^+$**	0.07 ± 0.01	(9.02 ± 0.52) x 10^1	(1.27 ± 0.14) x 10^3	12
**F475W$^+$**	0.08 ± 0.01	(6.01 ± 0.36) x 10^3	(8.08 ± 0.65) x 10^4	769
**R611A**	0.27 ± 0.02	(3.12 ± 0.43) x 10^3	(1.13 ± 0.92) x 10^4	108
**Y291A/H444A**	NA	NA	NA	NA

All assays were conducted at 25°C in 100 mM MES pH 6.0 and 150 mM NaCl. *indactes this is a specific activity and not a k_{cat} value. Data are technical triplicates and errors represent standard errors of the mean. NQ indicates data were not quantifiable using the spectrophotometric method employed. NA indicates no activity could be observed. $^+$ HA 70-130 kDa

Table S3. Melting temperature of BT4410^{PL33} and its mutants

Enzyme	T_m
**WT**	48.64 ± 0.20
**R103A**	46.44 ± 0.31
**H154A**	43.28 ± 0.20
**L174A**	46.11 ± 0.18
**W239A**	46.66 ± 0.16
**W242A**	47.68 ± 0.14
**W242V**	46.42 ± 0.31
**E286A**	49.15 ± 0.12
**Y291A**	49.28 ± 0.15
**H294A**	47.43 ± 0.24
**D301A**	44.80 ± 0.21
**R358A**	48.09 ± 0.09
**D388A**	42.63 ± 0.26
**H444A**	47.18 ± 0.06
**R611A**	48.80 ± 0.19
**Y291A/H444A**	47.33 ± 0.08
**Y470A**	47.45 ± 0.24
**Y470F**	44.83 ± 0.20
**F475A**	48.15 ± 0.12
**F475W**	42.65 ± 0.25

Differential scanning fluorimetry was performed to calculate protein melting temperatures and assays were conducted in 100 mM BTP pH 7.0 and 150 mM NaCl. Data are technical triplicates and errors shown as confidence intervals.

Table S4. Specific activity of *Bt*PL33^{HA,} *Bt*PL33$^{HA\text{-}loop}$, and *Bs*PL33^{heparosan} against select GAGs

Substrate	Specific activity (min^{-1})		
	Hyaluronic acid	Heparosan	Heparan sulfate
***Bt*PL33^{HA}**	(1.94 ± 0.80) x 10^3	NA	NA
***Bt*PL33$^{HA\text{-}loop}$**	0.44 ± 0.12	NQ	NQ
***Bs*PL33^{heparosan}**	NA	(2.89 ± 0.13) x 10^2	NQ

Enzymes were assays at 25°C by directly measuring double formation at A_{235nm}. Substrate concentrations deployed were 1 mg/ml for Hyaluronic acid, 0.1 mg/ml for heparosan, and 10 mg/ml for heparan sulfate. NA means no activity could be detected. NQ means the activity was not reliably measurable. For *Bt*PL33$^{HA\text{-}loop}$ against heparosan high enzyme concentrations interfered with the assay and the low availability of substrate prevented testing higher substrate concentrations with lower enzyme concentrations. The heparan sulfate batch proved incompatible with our assay as it had high readings at A_{235nm} combined with presumably a low number of cleavage sites as evidenced by TLC (Figure 10).

Table S5. Mutagenesis primers

Primer name	Primer (5' to 3')
R103A Forward	ACC GGT AAT GCC AAA ATC ATG GAA GTT CCC TAT
R103A Reverse	CAT GAT TTT GGC ATT ACC GGT CCG CTC ATA TTC
H154A Forward	CTG TCT GCT GCC CTG CCC CGG CAA AGC AGC AAA
H154A Reverse	CCG GGG CAG GGC AGC AGA CAG TAC CCA TGA ATT
L174A Forward	ATC ATT GAT GCC GGT TCC GGC GGA TAT GGT GCA
L174A Reverse	GCC GGA ACC GGC ATC AAT GAT CTG TTC GCG GAA
W239A Forward	ATC AAT AAC GCC AAT CCT TGG TGC AAC TCC AAT
W239A Reverse	CCA AGG ATT GGC GTT ATT GAT AAT CTC ACC CGG
W242A Forward	TGG AAT CCT GCC TGC AAC TCC AAT GCT TTG CAG
W242A Reverse	GGA GTT GCA GGC AGG ATT CCA GTT ATT GAT AAT
W242V Forward	TGG AAT CCT GTG TGC AAC TCC AAT GCT TTG CAG
W242V Reverse	GGA GTT GCA CAC AGG ATT CCA GTT ATT GAT AAT
E286A Forward	GGC GCT TGC GCC GAA GGG ACT TCT TAT TGG GGA
E286A Reverse	AGT CCC TTC GGC GCA AGC GCC GTC AGA CTT CAC
Y291A Forward	GGG ACT TCT GCC TGG GGA CAC GCG GCA GGT AAA
Y291A Reverse	GTG TCC CCA GGC AGA AGT CCC TTC TTC GCA AGC
H444 Forward	AAC GAA AGT GCC AAT CAT AAT GAT GTG GGA ACG
H444 Reverse	ATT ATG ATT GGC ACT TTC GTT ATT GAA TCC GCC
H294A Forward	TAT TGG GGA GCC GCG GCA GGT AAA CTT TAT GAT
H294A Reverse	ACC TGC CGC GGC TCC CCA ATA AGA AGT CCC TTC
D301A Forward	AAA CTT TAT GCC TAT TTG CAA ATA CTT TCT GAT
D301A Reverse	TTG CAA ATA GGC ATA AAG TTT ACC TGC CGC GTG
R358A Forward	CTG ATC TAT GCC TTT GGT AAA GCG GTA AAC AGC
R358A Reverse	TTT ACC AAA GGC ATA GAT CAG TAG AGG ATC GCC
D388A Forward	ATG GGA AAC GCC GCA TTC CGC TCG CTC CAG TCT
D388A Reverse	GCG GAA TGC GGC GTT TCC CAT GGT CGC GTA CGG

R611A Forward	GAC GAT CCG GCC CTC TCT AAT GTA TGG GGT AAG
R611A Reverse	ATT AGA GAG GGC CGG ATC GTC CAA TTG GAT TGT
Y470A Forward	GTA GGT ACA GCC ACT AAG CAG ACT TTC GGT AAA
Y470A Reverse	CTG CTT AGT GGC TGT ACC TAC CCC TGC ATC AAG
Y470F Forward	GTA GGT ACA TTC ACT AAG CAG ACT TTC GGT AAA
Y470F Reverse	CTG CTT AGT GAA TGT ACC TAC CCC TGC ATC AAG
F475A Forward	AAG CAG ACT GCC GGT AAA GAC CGT TAC ACC ATC
F475A Reverse	GTC TTT ACC GGC AGT CTG CTT AGT ATA TGT ACC
F475W Forward	AAG CAG ACT TGG GGT AAA GAC CGT TAC ACC ATC
F475W Reverse	GTC TTT ACC CCA AGT CTG CTT AGT ATA TGT ACC

Table S6. Crystallographic statistics table

**Collection statistics**	*Bt*PL33^{HA} Apo crystal form 1	*Bt*PL33^{HA} Apo crystal form 2	*Bt*PL33^{HA} Y291A HA dp4
Beamline	I-04	I-24	I-04
Date	13/04/19	26/09/21	29/07/22
Wavelength (Å)	1.65	0.99	0.95
Resolution (Å)	48.61-2.70 (2.83-2.70)	113.85-2.03 (2.17-2.03)	113.70- 1.80 (1.89-1.80)
Space group	$P22_12_1$	$P2_12_12_1$	$P2_12_12_1$
Unit-cell parameters			
a, b, c (Å)	74.51, 101.85, 162.87	53.53, 137.67, 202.51	53.64 137.30 202.84
α = β = γ (°)	90, 90, 90	90, 90, 90	90, 90, 90
No. of measured reflections	2202723 (291856)	431021 (19029)	836037(44403)
No. of independent reflections	34414(4486)	73386 (3670)	122182(6109)
Completeness (Spherical %)	100 (100)	75.0 (20.8)	87.1 (30.4)
Completeness (Ellipsoidal %)	-	90.8 (58.6)	95.4 (65.7)
Redundancy	31.4 (31.4)	5.9 (5.2)	6.8 (7.3)
$\langle I \rangle / \langle \sigma(I) \rangle$	15.8 (1.3)	6.6 (1.6)	14 (1.4)
CC(1/2)	0.999 (0.735)	0.990 (0.503)	0.999 (0.698)
**Refinement statistics***			
R_{work}/R_{free} (%)	18/28	19/25	18/22
No. of non-H atoms			
No. of protein, atoms	9885	9823	9900
No. of solvent atoms	2	333	700
No. of ligand atoms	2	2	129
r.m.s. deviation from ideal values			
Bond angle (°)	0.010	0.015	0.014
Bond length (Å)	2.10	2.54	2.22
Average B factor (Å^2)			
Protein	88.45	30.29	34.74
Solvent	49.39	30.91	39.15
Ligand	98.93	29.17	56.14
Ramachandran plot$^+$, most favoured regions (%)	95.0	96.5	95.8
Molprobity score	2.26	1.90	1.71
PDB code	8R75	8R6Z	8R70

Values in parenthesis are for the highest resolution shell. R_{free} was calculated using a set (5%) of randomly selected reflections that were excluded from refinement.

Table S7. Crystallographic statistics table

**Collection statistics**	*Bt*PL33^{HA} Y291A HA dp4	*Bt*PL33^{HA} Y291A HA dp4	*Bc*PL33^{HA} Apo crystal
Beamline	I-04	I0-4	I0-3
Date	11/08/22	11/08/22	23/03/23
Wavelength (Å)	1.22	1.33	0.98
Resolution (Å)	60.66-1.95 (1.95-1.92)	53.87-2.58 (2.66-2.58)	40.01-2.11 (2.17-2.11)
Space group	$P2_12_12_1$	$P2_12_12_1$	C2
Unit-cell parameters		53.87, 137.40, 203.33	
a, b, c (Å)	53.63, 137.37, 202.80	90, 90, 90	103.63, 91.39, 84.77
α = β = γ (°)	90, 90, 90		90, 109.28, 90
No. of measured reflections	757698 (26918)	317182 (30196)	288438 (25276)
No. of independent reflections	115460 (5594)	48609 (4374)	42387 (3507)
Completeness (Spherical %)	100 (99.9)	100 (100)	99.7 (100)
Completeness (Ellipsoidal %)	-	-	-
Redundancy	6.6 (4.8)	6.5 (6.9)	6.7 (7.2)
<I>/<σ(I)>	19.9 (1.5)	8.4 (1.5)	10.8 (1.5)
CC(1/2)	0.999 (0.824)	0.989 (0.574)	0.997 (0.705)
**Refinement statistics***			
R_{work}/R_{free} (%)	18/21	22/28	19/25
No. of non-H atoms			
No. of protein, atoms	9892	9821	4981
No. of solvent atoms	638	6	312
No. of ligand atoms	105	108	1
r.m.s. deviation from ideal values			
Bond angle (°)	0.009	0.007	0.008
Bond length (Å)	1.72	1.49	1.83
Average B factor (Å^2)			
Protein	37.33	48.50	42.87
Solvent	39.06	44.22	47.49
Ligand	47.47	75.47	41.60
Ramachandran plot^{+}, most favoured regions (%)	97.5	94.65	96.8
Molprobity score	1.26	1.79	1.46
PDB code	8R71	8R72	8R73

Values in parenthesis are for the highest resolution shell. R_{free} was calculated using a set (5%) of randomly selected reflections that were excluded from refinement.

Dataset S1 (separate file). Environmental isolation of PL33_1 sequences

Dataset S2 (separate file). Environmental isolation of PL33_2 sequences

Dataset S3 (separate file). Raw absorbance value of HPAEC runs determining degrees of processivity

Dataset S4 (separate file). R.M.S.D. analysis of BTPL33^{HA} versus orthologous protein structures from distantly related families. Analysis was performed using PDBeFold.

SI References

1 Kabsch, W. Xds. *Acta crystallographica. Section D, Biological crystallography* **66**, 125-132 (2010). https://doi.org:10.1107/S0907444909047337

2 Evans, P. Scaling and assessment of data quality. *Acta crystallographica. Section D, Biological crystallography* **62**, 72-82 (2006). https://doi.org:10.1107/S0907444905036693

3 Evans, P. R. An introduction to data reduction: space-group determination, scaling and intensity statistics. *Acta crystallographica. Section D, Biological crystallography* **67**, 282-292 (2011). https://doi.org:10.1107/S090744491003982X

4 Emsley, P., Lohkamp, B., Scott, W. G. & Cowtan, K. Features and development of Coot. *Acta crystallographica. Section D, Biological crystallography* **66**, 486-501 (2010). https://doi.org:10.1107/S0907444910007493

5 Murshudov, G. N. *et al.* REFMAC5 for the refinement of macromolecular crystal structures. *Acta crystallographica. Section D, Biological crystallography* **67**, 355-367 (2011). https://doi.org:10.1107/S0907444911001314

6 Chen, V. B. *et al.* MolProbity: all-atom structure validation for macromolecular crystallography. *Acta crystallographica. Section D, Biological crystallography* **66**, 12-21 (2010). https://doi.org:10.1107/S0907444909042073

7 Lebedev, A. A. *et al.* JLigand: a graphical tool for the CCP4 template-restraint library. *Acta crystallographica. Section D, Biological crystallography* **68**, 431-440 (2012). https://doi.org:10.1107/S090744491200251X

8 Collaborative Computational Project, N. The CCP4 suite: programs for protein crystallography. *Acta crystallographica. Section D, Biological crystallography* **50**, 760-763 (1994). https://doi.org:10.1107/S0907444994003112

9 Byrne, D. P., London, J. A., Eyers, P. A., Yates, E. A. & Cartmell, A. Mobility shift-based electrophoresis coupled with fluorescent detection enables real-time enzyme analysis of carbohydrate sulfatase activity. *The Biochemical journal* **478**, 735-748 (2021). https://doi.org:10.1042/BCJ20200952

10 Drula, E. *et al.* The carbohydrate-active enzyme database: functions and literature. *Nucleic Acids Res* **50**, D571-D577 (2022). https://doi.org:10.1093/nar/gkab1045

11 Li, W. & Godzik, A. Cd-hit: a fast program for clustering and comparing large sets of protein or nucleotide sequences. *Bioinformatics* **22**, 1658-1659 (2006). https://doi.org:10.1093/bioinformatics/btl158

12 Katoh, K., Rozewicki, J. & Yamada, K. D. MAFFT online service: multiple sequence alignment, interactive sequence choice and visualization. *Brief Bioinform* **20**, 1160-1166 (2019). https://doi.org:10.1093/bib/bbx108

13 Capella-Gutierrez, S., Silla-Martinez, J. M. & Gabaldon, T. trimAl: a tool for automated alignment trimming in large-scale phylogenetic analyses. *Bioinformatics* **25**, 1972-1973 (2009). https://doi.org:10.1093/bioinformatics/btp348

14 Guindon, S. & Gascuel, O. A simple, fast, and accurate algorithm to estimate large phylogenies by maximum likelihood. *Syst Biol* **52**, 696-704 (2003). https://doi.org:10.1080/10635150390235520

15 Guindon, S. *et al.* New algorithms and methods to estimate maximum-likelihood phylogenies: assessing the performance of PhyML 3.0. *Syst Biol* **59**, 307-321 (2010). https://doi.org:10.1093/sysbio/syq010

16 Letunic, I. & Bork, P. Interactive Tree Of Life (iTOL) v5: an online tool for phylogenetic tree display and annotation. *Nucleic Acids Res* **49**, W293-W296 (2021). https://doi.org:10.1093/nar/gkab301

17 Waterhouse, A. M., Procter, J. B., Martin, D. M., Clamp, M. & Barton, G. J. Jalview Version 2--a multiple sequence alignment editor and analysis workbench. *Bioinformatics* **25**, 1189-1191 (2009). https://doi.org:10.1093/bioinformatics/btp033

18 Crooks, G. E., Hon, G., Chandonia, J. M. & Brenner, S. E. WebLogo: a sequence logo generator. *Genome Res* **14**, 1188-1190 (2004). https://doi.org:10.1101/gr.849004

19 Manalastas-Cantos, K. *et al.* ATSAS 3.0: expanded functionality and new tools for small-angle scattering data analysis. *J Appl Crystallogr* **54**, 343-355 (2021). https://doi.org:10.1107/S1600576720013412

20 Schneidman-Duhovny, D., Hammel, M., Tainer, J. A. & Sali, A. FoXS, FoXSDock and MultiFoXS: Single-state and multi-state structural modeling of proteins and their complexes based on SAXS profiles. *Nucleic Acids Res* **44**, W424-429 (2016). https://doi.org:10.1093/nar/gkw389

21 Mirdita, M. *et al.* ColabFold: making protein folding accessible to all. *Nat Methods* **19**, 679-682 (2022). https://doi.org:10.1038/s41592-022-01488-1

22 Steinegger, M. & Soding, J. MMseqs2 enables sensitive protein sequence searching for the analysis of massive data sets. *Nat Biotechnol* **35**, 1026-1028 (2017). https://doi.org:10.1038/nbt.3988

23 Varadi, M. *et al.* AlphaFold Protein Structure Database: massively expanding the structural coverage of protein-sequence space with high-accuracy models. *Nucleic Acids Res* **50**, D439-D444 (2022). https://doi.org:10.1093/nar/gkab1061

24 Van Der Spoel, D. *et al.* GROMACS: fast, flexible, and free. *J Comput Chem* **26**, 1701-1718 (2005). https://doi.org:10.1002/jcc.20291

25 Bjelkmar, P., Larsson, P., Cuendet, M. A., Hess, B. & Lindahl, E. Implementation of the CHARMM Force Field in GROMACS: Analysis of Protein Stability Effects from Correction Maps, Virtual Interaction Sites, and Water Models. *J Chem Theory Comput* **6**, 459-466 (2010). https://doi.org:10.1021/ct900549r

26 de Groot, B. L. *et al.* Prediction of protein conformational freedom from distance constraints. *Proteins* **29**, 240-251 (1997). https://doi.org:10.1002/(sici)1097-0134(199710)29:2<240::aid-prot11>3.0.co;2-o
